# A Scoping Review of Observational Studies Examining Relationships between Environmental Behaviors and Health Behaviors

**DOI:** 10.3390/ijerph120504833

**Published:** 2015-05-05

**Authors:** Jayne Hutchinson, Stephanie L. Prady, Michaela A. Smith, Piran C. L. White, Hilary M. Graham

**Affiliations:** 1 Department of Health Sciences, University of York, Heslington York YO10 5DD, UK; E-Mails: stephanie.prady@york.ac.uk (S.L.P.); mas580@york.ac.uk (M.A.S.); hilary.graham@york.ac.uk (H.M.G.); 2 Environment Department, University of York, Heslington York YO10 5DD, UK; E-Mail: piran.white@york.ac.uk

**Keywords:** lifestyle, pro-environmental behavior, health-related behavior, active travel, public transport use, physical activity, consumption

## Abstract

Individual lifestyles are key drivers of both environmental change and chronic disease. We undertook a scoping review of peer-reviewed studies which examined associations between environmental and health behaviors of individuals in high-income countries. We searched EconLit, Medline, BIOSIS and the Social Science Citation Index. A total of 136 studies were included. The majority were USA-based cross-sectional studies using self-reported measures. Most of the evidence related to travel behavior, particularly active travel (walking and cycling) and physical activity (92 studies) or sedentary behaviors (19 studies). Associations of public transport use with physical activity were examined in 18 studies, and with sedentary behavior in one study. Four studies examined associations between car use and physical activity. A small number included other environmental behaviors (food-related behaviors (n = 14), including organic food, locally-sourced food and plate waste) and other health behaviors ((n = 20) smoking, dietary intake, alcohol). These results suggest that research on individual environmental and health behaviors consists largely of studies examining associations between travel mode and levels of physical activity. There appears to be less research on associations between other behaviors with environmental and health impacts, and very few longitudinal studies in any domain.

## 1. Introduction

Individual lifestyles, and the wider social and economic systems that sustain them, are key drivers of environmental change and chronic disease. The earth’s climate and ecosystems are changing as a result of economies and lifestyles sustained by consumption of natural resources, including fossil fuels, tropical forests and other natural habitats, water systems and fish stocks [1,2,3]. First established in early-industrialising and high-income countries of North America and Europe, these modern ways of living are increasingly the global norm. Modern lifestyles also lie behind the non-communicable diseases—heart disease, stroke, cancer, diabetes and chronic respiratory disease—that account for the majority of premature deaths in high-income countries and, increasingly, worldwide [4,5,6]. 

However, the urgency of the environmental and health challenges facing humanity is bringing these fields together [2,7]. Evidence to inform policies to promote environmental sustainability and public health requires information at all spatial scales, from the local to the global. One key component of this evidence base is information on individual behaviors with significant environmental and health impacts. 

The scientific community is responding to this need. The major focus to date has been on travel-related behavior, with systematic reviews bringing together evidence on active travel (walking and cycling for journeys) and physical activity [8,9,10,11], and public transport use and physical activity [12]. However, other behaviors have both environmental and health effects. For example, unhealthy diets (high in animal fat and processed products and low in fruit, vegetables and fibre) are typified by high environmental costs [13,14]; conversely, vegetarian, organic and locally-sourced diets can bring both health and environmental co-benefits [14,15]. Beyond travel and diet, individual lifestyles are made up of a broader spectrum of everyday practices with environmental and health effects, including cigarette smoking, waste management (including food waste), and household energy conservation and use [16,17]. Evidence on these patterns would, in turn, facilitate the integration of quantitative studies of behavior with the broader swathe of social scientific research on the psychosocial influences on every day practices, including lifestyle types, self-identity and habit [18,19,20].

Evidence on whether and how behaviors with environmental and health impacts are associated is also important for policies seeking to promote lifestyles with environmental and health co-benefits. However beyond the focused reviews noted above, there has been, to our knowledge, no broader review of studies investigating associations between environmental and health behaviors. 

We therefore undertook a scoping review of this important area of research. Scoping reviews provide an initial overview of a research field, and are regarded as particularly suitable for research spanning disciplines and methods. They therefore employ broad search terms and do not apply quality filters [21,22,23]. 

Our scoping review aims to determine the extent and breadth of research investigating associations between environmental and health behaviors of adults and children living in high income countries. As part of this process, we note broad findings from the studies.

## 2. Methods

### 2.1. Search

We searched EconLit, Medline, Medline in process, BIOSIS (1 January 1969 to 3 January 2014) and the Social Science Citation Index in January 2014 without date limitations. We included a broad range of terms relevant to individual health and environmental behavior including: lifestyle, health behavior, pro-environmental behavior, recycling, water and energy conservation, sustainable agriculture, locally grown, allotment, diet, smoking, drug abuse, alcohol, physical activity, walking and various terms for active travel and public transport (see online supplementary materials for full search strategy).

We refer to papers that report analyses of empirical research as ‘studies’. Multiple studies can therefore be based on the same survey or dataset, for example using different behavioral measures. 

### 2.2. Inclusion and Exclusion Criteria

We included observational studies where data were collected on behaviors of individuals living in community (non-institutional) settings in one or more member states of the Organisation for Economic Co-operation and Development (OECD). We also included non-experimental studies in these settings that evaluated the impact of a policy change or local development. 

We included studies that examined the bivariate association between an environmental and a health behavior in individuals. One of these behaviors could be a ‘dual behavior’ with impacts on both the environment and on health, like active travel or consumption of organic food [24,25]. The health behavior or the environmental behavior could be either the predictor or outcome variable. There were also no restrictions on whether or how analyses were adjusted. We excluded studies which were experimental in design, were not peer reviewed, were not written in English, or where the unit of measurement was an organization, for example, the energy conservation behaviors of companies and the food waste of a school.

We included systematic reviews of observational studies where the review aimed to examine the relationship between an environmental and a health behavior. There were two reasons for including systematic reviews: to determine whether the literature in this area had already been systematically collated; and to assess whether our search terms were sufficiently sensitive to identify the majority of relevant studies of interest, in order to judge the generalizability of our findings. We excluded non-systematic literature reviews.

### 2.3. Screening Data Extraction

**Figure 1:**
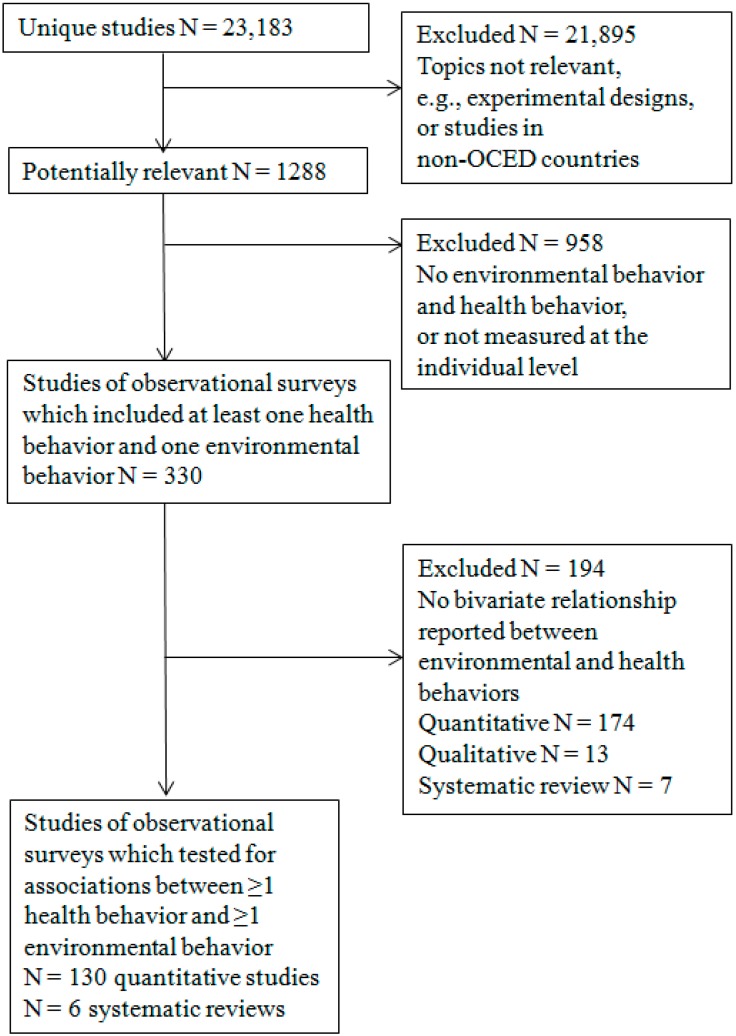
Flow chart of extracted and excluded studies.

After removing duplicates, citations that did not meet the inclusion criteria by the title or abstract were removed by one author (Stephanie L. Prady). Full papers of the 1288 potentially relevant articles were screened by one author (Stephanie L. Prady) and checked by another (Jayne Hutchinson) for those that included both a health behavior and an environmental behavior (Figure 1). The 330 articles that met this initial inclusion criterion were independently screened by two authors (Stephanie L. Prady/Jayne Hutchinson or Michaela A. Smith/Jayne Hutchinson) for full inclusion. Those without quantitative data on associations between environmental and health behaviors were excluded. Of these 330 articles, six were systematic reviews.

Relevant information from included studies was extracted by one author (Jayne Hutchinson or Michaela A. Smith) onto a piloted form and the extracted information checked by another author (Stephanie L. Prady or Jayne Hutchinson). Discrepancies were resolved within the project team by consensus. 

### 2.4. Synthesis

We grouped the studies by the types of behavior they analysed, and summarised the type of settings, populations and study designs for each group, along with the measures used and broad findings. Where we identified a systematic review, we counted the number of studies included in the review that potentially met our inclusion criteria and noted how many studies had been missed by our search. 

## 3. Results

### 3.1. Summary of the Characteristics of the Studies

We included 136 studies in our scoping review; 130 research studies which reported the association between environmental and health behaviors using data from 109 surveys or primary datasets (Table 1 and supplementary materials Table S1); and also six systematic reviews on the topic (supplementary materials Table S2). 

The earliest study was published in 1991; the remaining 129 were all published since 2003. Over half (55%) were published from 2009–2013, pointing to a recent and rapid increase in research. Most studies drew on surveys conducted in USA (28%), UK (18%), Australia (13%), Canada (8%) and Denmark (6%). Just over half of the studies related to children and young people (54%), and only two studies related to adults of a senior age.

Studies of active travel with one or more health behaviors predominated and 74% examined relationships between active travel and physical activity (n = 92) and/or sedentary behavior (n = 19). There were also a larger number of studies on public transport use and physical activity (n = 18). Eight studies examined the relationship between active travel and smoking, four of which also examined relationships with active travel and alcohol use and/or diet. We found few studies of non-travel related environmental behaviors, with very few on food-related behaviors and none on home energy behaviors. Eight studies examined the relationship between organic food use and another health or environmental behavior. 

Most studies relied on self-reported data (adults) and parent-reported data (children). The exceptions were studies involving physical activity and sedentary behavior where 40% of the studies used objective measures, for example pedometers or accelerometers. Plate waste studies also included objective weight-based measures. 

Nearly all the studies were cross-sectional (n = 123), with only seven reporting the results of longitudinal or pre-post analyses. Results from the review are described in the sections below. The numbers of studies are summarized in Table 1 and their details are summarized in the supplementary materials in Tables S1 and S2. 

### 3.2. Active Travel 

#### 3.2.1. Active Travel and Physical Activity

Ninety-two studies examined associations between active travel and physical activity [26,27,28,29,30,31,32,33,34,35,36,37,38,39,40,41,42,43,44,45,46,47,48,49,50,51,52,53,54,55,56,57,58,59,60,61,62,63,64,65,66,67,68,69,70,71,72,73,74,75,76,77,78,79,80,81,82,83,84,85,86,87,88,89,90,91,92,93,94,95,96,97,98,99,100,101,102,103,104,105,106,107,108,109,110,111,112,113,114,115,116,117] (summarized in Table 1 and listed as category AT/PA in Table S1 in the online supplementary materials). The majority were from the UK (n = 24) and USA (n = 19), followed by Australia (n = 12), Canada (n = 7), Denmark (n = 6) and New Zealand (n = 5). Other countries with less than five studies included: Norway, Netherlands, Belgium, Switzerland, Spain, Portugal, Estonia, Germany, Sweden, and Ireland. The majority were conducted among children (70%, n = 64), including 8 studies that included only children under 10 years old. Only two studies reported analyses of only adults over 65 [46,87]. 

The vast majority of studies used cross-sectional analyses—only six studies out of 92 (6.5%) reported results from longitudinal or pre/post analysis to examine associations between active travel and physical activity [29,41,81,99,102,109]. Just over half of the studies (n = 48) used objective measures (e.g., accelerometer, pedometer) to assess physical activity (see supplementary Table S1).

Overall, most studies (n = 72; 78%, representing 75% of the children’s and 86% of the adults’ studies) reported a positive association between active travel and physical activity; however many of these (n = 32) reported mixed results overall (e.g., when using more than one measure of physical activity, or in sub-analyses such as for gender). Of the 20 studies that reported no association, 12 used objective measures to assess physical activity. The average numbers of participants in these 20 studies were much lower than in studies which did report an association. This may be indicative of insufficient power to find associations. 

We found four systematic reviews examining active travel and physical activity, all of which were published between 2008 and 2013 [8,9,10,11]. They included seven studies that were missed by our search but appeared to meet our inclusion criteria. Three reviews focused on children (<18 years old) and one review focused on adults (18+). Among the systematic reviews that focused on children, the number of included studies ranged from 13 [8], to 42 [10], depending on the year of the review and how narrowly active travel and physical activity were defined in the inclusion criteria. All three reviews involving children mostly reported associations between active travel and physical activity. In the review which focused on adults [11], the relationship between active travel and physical activity was more mixed, with only five of 15 studies reporting positive associations in the expected direction. 

#### 3.2.2. Active Travel and Sedentary Behaviors

The 19 studies on active travel and sedentary behaviors were carried out in a range of countries: USA, UK, Australia, Canada, Poland, Belgium, Germany, Portugal and the Netherlands. Six studies were on adults [33,54,112,118,119,120], and 13 on children aged between 5 to 18 years old [32,52,67,70,74,79,88,92,96,102,115,121,122]. 

Sedentary time was objectively measured by accelerometer in only four of the studies [70,88,92,102], and these all related to children. In the majority of the studies, individuals were asked to self-report the number of hours they spent watching TV, using the computer or gaming. Active travel was self-reported in all the studies, and distance travelled was measured objectively in one study [74]. Only one study used longitudinal analyses [102].

The majority of the 19 studies reported no associations between active travel and sedentary behaviors. Objectively measured sedentary behavior was inversely associated with active travel in only one of the studies [70]. This study of seven year olds used a higher cut-off for sedentary time of <1100 counts per minute (cpm) of activity measured by accelerometer compared to <100 cpm used in the other three studies [88,92,102]. There was no evidence from one study that desk jobs had an association with cycling to work [54], but two other studies found that people in sitting occupations were significantly less likely to travel actively that those in more active occupations [33,118].

The combined time of self-reported total screen time was not associated with active travel in any study of adults or children [79,115,121,122]. Some studies analyzed each sedentary behavior separately [52,74,119], including reading and computer use, whereas some only reported TV viewing [32,54,88]. In the analyses of separate sedentary behaviors, only two studies found an association with active travel [32,52], both were studies of children, and behaviors were reported by parents. Children’s TV viewing was positively associated with always cycling to or from school in one study [52], and in another study, children watching over three hours of TV a day were nearly 30% more likely to walk to school than those who watched less [32]. Conversely, in two studies of adults, TV watching was inversely associated with active travel [54,119]. 

We found one systematic review on active travel and sedentary activity [10]. It reported five studies relating to children [67,70,74,88,92], all of which had been identified through our search. Overall its findings were inconclusive. Our review found eight further studies on children in this topic area, only one of which reported an association as mentioned above [32]. Overall our review did not point to an association between active travel and measured or specific sedentary behaviors; however, as noted in the Methods section, we did not restrict studies to those where findings were reported post adjustments for confounders. 

#### 3.2.3. Active Travel and Other Health Behaviors 

##### Smoking

The relationship between active travel and smoking was examined in eight studies; these were from Canada [33,96,122], Germany [74], Finland [123], Poland [71,72], and the USA [60]. Three involved children [74,96,122]. Five involved adults [33,60,71,72,123]; four of these adult studies were nationally representative surveys [33,60,71,72]. Active travel and smoking behavior were reported by questionnaire, and all were cross-sectional.

Overall, results were mixed in this small number of studies on active travel and smoking. For example in the Polish and Finnish studies of adults, smoking was inversely associated with active travel among men, but not women [71,72,123]. However in the American study, current smokers were more likely to travel actively [60]. In the large Canadian survey, smoking was not associated with active travel in general, but was found to be associated with walking over six hours a week for a purpose (*i.e*., to work, school or an errand) [33]. 

With respect to children, active travel was inversely associated with smoking in boys but not girls in a small study of German 14 year olds [74]. In two Canadian studies of children, smoking was inversely related to active travel in the larger [96], but not in the smaller study [122]. 

**Table 1:** Summary of number of observational studies that analysed the association between health behavior and environmental behaviors.

Domain	Active Travel & Physical Activity	Active Travel & Sedentary Behaviors	Active Travel & Other Behaviors	Public Transport Use & Physical Activity	Public Transport Use & Sedentary Behaviors	Public Transport & Other Behaviors	Car Use (Only) & Physical Activity	Car Use (Only) & Other Behaviors	Recycling & Other Sedentary Behavior	Organic Food Use & Other Behaviors	Locally Sourced Food & Health Behaviors	Plate Waste & Health Behaviors
Primary Studies	92	19	10	18	1	1	3	1	1	8	5	2
Published before 2010	40	9	4	8	−	−	1	−	1	2	1	2
Published 2010 onwards	52	10	6	10	1	1	2	1	−	6	4	−
Children only	64	13	5	1	1	−	1	−	−	−	1	2
Adults/ mixed **^a^**	28	6	5	17	−	1	2	1	1	8	4	−
USA	19	5	1	10	−	1	−	−	1	2	4	1
Canada	7	3	3	1	−	−	2	−	−	−	−	−
Australasia	17	−	1	3	−	−	2	−	−	−	1	−
UK	24	3	1	2	1	−	−	−	−	−	−	−
Scandinavia **^b^**	10	1	1	−	−	−	−	1	−	5	−	−
Other European	17	5	3	2	−	−	−	−	−	1	−	−
Other OECD	−	−	−	−	−	−	−	−	−	−	−	1
Behaviour(s) measured objectively **^c^**	45	4	1	5	1	−	−	−	−	−	−	2
Longitudinal analyses	6	1	−	1	−	−	−	−	−	−	1	2
Reference numbers	[26,27,28,29,30,31,32,33,34,35,36,37,38,39,40,41,42,43,44,45,46,47,48,49,50,51,52,53,54,55,56,57,58,59,60,61,62,63,64,65,66,67,68,69,70,71,72,73,74,75,76,77,78,79,80,81,82,83,84,85,86,87,88,89,90,91,92,93,94,95,96,97,98,99,100,101,102,103,104,105,106,107,108,109,110,111,112,113,114,115,116,117]	[32,33,52,54,67,70,74,79,88,92,96,102,112,115,118,119,120,121,122]	[33,56,57,60,71,72,74,96,122,123]	[46,73,84,92,95,124,125,126,127,128,129,130,131,132,133,134,135,136]	[92]	[137]	[138,139,140]	[141]	[120]	[142,143,144,145,146,147,148,149]	[145,150,151,152,153]	[155,156]
Systematic reviews	4 [8,9,10,11]	1 [10]		1 [12]							1 [154]	

Notes: ^**a**^ Some studies in the “Adults/mixed” column may include children as well as adults; ^**b**^ Finland, Denmark, Norway and Sweden; ^**c**^ Physical activity was objectively measured in most studies that used objective measures; most of these studies also used self-reported measures for physical activity; some studies are included in more than one domain of health and environmental behavior.

##### Alcohol Use and Diet

Four studies from Poland, Germany, Finland and Canada examined active travel and alcohol use [72,74,122,123]. Four studies from New Zealand, England, Poland and Germany examined active travel and diet [56,57,72,74]. Four analyzed data from children [56,57,74,122] and two from adults [72,123]. All analyses were cross-sectional and used self-reported or parental reported data. 

Overall, results were mixed in this small number of studies on active travel and alcohol intake. In the Finnish study the highest percentage of drinkers were found in the middle active travel group for both men and women (1–29 min per day) [123]. However, there was no evidence of significant associations between active travel and alcohol consumption, or dietary patterns in studies of Polish adults or German children [72,74]. Calorie intake in Polish male active travellers was slightly higher than non-active travellers [72]. Additionally no difference in estimated dietary intake was found between groups of UK children who walk to primary school and those who travelled by car [57]. There was no significant difference in fruit and vegetable intake between New Zealand boys in a cluster characterized by active travel and two other clusters determined from time use data on physical activity and diet [56].

### 3.3. Public Transport Use 

#### 3.3.1. Public Transport Use and Physical Activity

The majority of the 18 studies that examined the relationship between public transport use and physical activity [46,73,84,92,95,124,125,126,127,128,129,130,131,132,133,134,135,136] were undertaken in the USA [73,84,124,127,129,130,131,132,133,136]. Three were carried out in Australia [95,125,134], two in the UK [46,92], and one each in Switzerland [126], Canada [135], and Germany [128]. Only one study involved only children [92], and one involved only older adults over 70 years of age [46]. One was a before-and-after study [132]; the rest used cross-sectional analyses. Five used objectively measured physical activity [46,92,129,134,136].

The majority of studies reported a positive association between public transport use and physical activity among adults. In the one study of children, those who reported traveling to school by public transport had similar levels of activity to those who walked [92]. All but one [132] of the 17 studies of adults reported a significant positive association between public transport use and physical activity. Ten studies reported significant associations between public transport use and time spent walking or in moderate exercise, or distance walked [46,84,124,125,127,129,130,134,135,136]. Five of these studies assessed physical activity objectively, three studies objectively measured steps taken [46,134,136], and two measured minutes of moderate or moderate to vigorous physical activity using pedometers and accelerometers [46,129]. The largest study of 28,771 individuals estimated 8–10 min of additional walking time from self-reported data [127]. One study found that public transport users had higher levels of walking or bicycling outside of commuting compared with car users, but not higher levels of other non-commute exercise [84]. 

Three out of the five adult studies that explored whether public transport use helped meet physical activity recommendations found that they did [95,124,130,132,136]. One cross-sectional analysis showed no association [136], and one found no change in physical activity after the installation of a light railway line [132]. Studies that examined frequency of walking [73,131], or bicycle use [133], in relation to public transport use all reported positive associations. One German study found a negative association between rail transport use and bicycling for any purpose [128]. Another study focused specifically on holiday-makers and found positive associations between travelling by train to the holiday destination and sports activity and cycling for transport whilst at the holiday destination [126].

We found a systematic review on public transport use and physical activity [12]. Seven out of the eight non-experimental studies in the systematic review were included in our scoping review. From the results of nine studies, it reported that between 8–33 additional minutes of walking time was attributable to public transport use. Minutes walked were self-reported in the majority of studies in the review. 

#### 3.3.2. Public Transport Use and Sedentary Behavior

Only one study examined associations between public transport use and sedentary behavior. This was a cross-sectional study of English children aged 9–10 years old, which objectively measured physical activity [92]. There was no evidence of associations between these behaviors in this study.

#### 3.3.3. Public Transport Use and Other Health Behaviors (Diet) 

Only one study examined associations between public transport use and other health behaviors, *i.e.*, diet. This was a cross-sectional study of USA adults that used self-reported data [137]. No difference in self-reported fruit and vegetable consumption was found between car, public transport and multimodal transport users.

### 3.4. Car Use

#### 3.4.1. Car Use and Physical Activity

This section includes studies that have compared physical activity of car users with non-car users. Studies that compared physical activity of car users with that of active travellers or public transport users have been discussed in previous sections. Three cross-sectional studies using self-reported behavior examined the relationship between car use or non-car use and physical activity; two were based in Canada [138,139] and one in Australia [140]. Two examined adults [139,140] and one examined children as passengers [138]. Not meeting physical activity recommendations was significantly related to car use in the Australian survey of adults [140], and over 1680 min per week of car use in the study of Canadian adults [139]. Nevertheless, children transported by parents by car so they could participate in sports 5–7 days a week were more likely to meet physical activity recommendations [138]. 

#### 3.4.2. Car Use and Other Health Behaviors (Smoking)

We found only one study which examined associations between car use and smoking status. This was a longitudinal study of Swedish adults aged 30–60 that used self-reported data [141]. Only a very weak correlation was found between increased car use and smoking.

### 3.5. Other Environmental Behaviors

#### 3.5.1. Recycling and Sedentary Behavior

Only one study examined recycling and sedentary behavior; this was a cross-sectional analysis of US adults using self-reported data [120]. Hours of newspaper reading was associated with recycling newspapers, but not with donating furniture or clothes; there was no evidence of associations between these recycling behaviors and hours spent watching TV or reading magazines. 

#### 3.5.2. Organic Food Use and Other Health or Environmental Behaviors

Eight studies examined relationships between organic food purchase or consumption and another health or environmental behavior (e.g., fruit and vegetable intake, nutrient levels, physical activity, smoking, alcohol intake, local food shopping, pro-environmental purchasing, recycling, public transport use or active travel) [142,143,144,145,146,147,148,149]. Two were undertaken in the USA [144,145], two in Denmark [146,147], two in Norway [148,149], one in Sweden [143], and one in France [142]. All involved cross-sectional analyses of adults, using self-reported data. 

Overall, in this small number of studies, organic food use was associated with increased pro-environmental behaviors and increased fruit and vegetable intake, but associations with some unhealthy behaviors were also found. 

Compared to non-organic users, organic food use was linked to greater consumption of fruit and vegetables or increased nutrient levels or other healthful dietary factors in all four analyses of large surveys that examined this relationship [142,146,148,149]. Two studies analyzed the same Norwegian survey of pregnant women using different methods [148,149]. Two studies which measured energy intake found significant associations between increased energy intake and organic food use [146,149]. Vegetarians/vegans were reported to be between 19 and 23 times more likely to be organic food users than non-vegetarians [146,149]. 

Two large studies of pregnant women reported associations between organic food use and increased physical activity; associations were found between organic food use and smoking status [146,148]. Additionally, one of the studies reported associations between organic food consumption and increased drinking during pregnancy [148]. 

Four studies reported positive relationships between pro-environmental behaviors and organic food use [143,144,145,147]. Organic food use was linked to local food shopping, specifically farmers’ markets and following a special diet [145], and purchasing bio-degradable or refillable products [144]. Additionally, organic food use was significantly associated with recycling and using public transport or bicycle as alternative forms of transport [147], and associated with advanced recycling and a group of other pro-environmental behaviors [143]. 

#### 3.5.3. Locally Sourced Food and Health Behaviors

We found five studies in this category [145,150,151,152,153]; four from the USA [145,151,152,153] and one from Australia [150]. All involved cross-sectional analyses of adults using self-reported data.

Overall in this small number of studies, purchase of locally sourced food was associated with healthier behaviors. As noted in the sub-section above, organic food consumption has also been linked to locally-sourced food [145].

The use of locally sourced food, either by participation in community gardening or via a community supported agriculture scheme, was associated with increased fruit and vegetable intake in two small USA studies [151,152]. Additionally, purchasing food products directly from producers was associated with greater daily physical activity in another small USA study [153]. Furthermore, Australian Aboriginals with high scores on a caring for the country index were more likely to exercise and consume bush food than those with low scores; this pro-environmental behavior was not associated with smoking and alcohol use [150]. 

One systematic review reported on the positive association between good local availability (e.g., access to one’s own vegetable garden, having low food insecurity) and increased fruit or vegetable intake [154].

#### 3.5.4. Plate Waste and Health Behaviors 

Two studies examined the relationship between objectively measured plate waste and diet or objectively measured physical activity in school children living in Korea [155] and USA [156]; these used cross-sectional analyses. 

Increased plate waste in school children was linked to indicators of poorer diet in the two studies found. In the Korean study, children with habitual plate waste were less likely to eat a variety of vegetables [155] than those without habitual plate waste, and their intake of folic acid, a key nutrient, was lower (and below UK Estimated Average Requirements). In the US study, children who purchased competitive food items (including sweet and salty snacks) with lunch wasted more fruit, meat and grain products but not significantly more vegetables [156]. No association was found between plate waste and physical activity [155].

## 4. Discussion

Our scoping review provides an overview of an emerging area of research at the interface between environmental sustainability and public health. It searched for observational studies of environmental and health behaviors of adults and children conducted in high-income countries with no date restriction. A large number of studies were identified (130 primary analyses and six systematic reviews). 

Studies analyzing active travel with one or more health behavior predominated; the majority examined relationships between active travel and physical activity (n = 92) and/or sedentary behavior (n = 19). There were also a large number of studies on public transport use and physical activity (n = 18). However, only nine studies examined travel-related behaviors with health behaviors such as smoking, alcohol intake or diet. Few studies examined other associations between environmental and health behaviors, including domestic energy use.

### 4.1. Strengths and Weaknesses of the Review

The original focus and the breadth of this review are two of its major strengths. We adopted a systematic approach to the identification of potentially relevant papers, including searching many research databases and using two reviewers to screen and extract information from studies. Some limitations of our review should be noted. Like other scoping reviews [21,22], it applied no quality filters; however, as it only included studies published in peer-reviewed journals, a de facto quality filter was used. As an initial overview of research, our review reports broad findings only and we did not report which analyses had been adjusted. However, we provide information on the design of each of the studies in our review (Table S1 in the supplementary materials). 

The broad search strategy may have resulted in relevant studies being missed. We consider the search terms worked well for travel behavior, physical activity and sedentary behavior. For instance, we located studies additional to those identified in recent systematic review of active travel and sedentary behavior [10]. 

For food-related behavior, a particularly broad set of behaviors with a wide set of descriptors, the search terms may have missed more studies. For example, we are aware the search missed studies with evidence on use of farmers’ markets and community gardens (environmental behaviors) and diet (a health behavior). Thus, our search did not find articles reviewed by McCormack, *et al.* [157] relating to the nutritional implications on adults of using farmers’ markets and community gardens: six non-intervention studies in this review examined associations with fruit and vegetable intake. Additionally, our search did not find studies on gardening or conservation work and physical activity (e.g., [158]). We included a range of relevant food-related terms (e.g., organic food, locally grown, ecological consumption, allotment), but did not include vegetarian or meat consumption among our search terms, and are therefore likely to have missed studies of a dual environmental/health behavior [13,24]. While our search terms may have been insufficiently extensive, our experience with this review suggests that the complex area of food-related behaviors with environmental and health impacts requires a separate and focused review. Given the centrality of food to the environment-energy-health nexus [159], a review of this area is an important priority for future research.

### 4.2. Key Findings

Our review provides evidence that the research community is responding to the need for evidence on behaviors with both environmental and health impacts. Studies with this dual focus represent a recent and expanding field of research. We found only one study published before 2003 [120]. Since then, research has increased rapidly, rising from two studies in each of 2003 and 2004 to 15 plus in each of the years from 2009. Children and adults are represented across the studies but only two focused on older age groups. The majority of studies were conducted in the USA, Australia, Canada and the UK. However, findings are likely to be relevant to other high-income countries with lifestyles that underlie the global chronic disease epidemic [5,160] and have contributed most to climate and environmental change [1,2,3,7]. 

Secondly, almost all the evidence derives from cross-sectional studies. The current evidence base can therefore shed little light either on the determinants of associations between environmental and health behaviors or on changes in these associations across the life course. The dearth of longitudinal studies is one of the major gaps for future research identified by this, and other, reviews [8,9,11,154]. Further reviews of experimental and qualitative studies, as well as quantitative studies, may also be useful for examining causal pathways and lived experience underpinning behaviors and lifestyle choices, including those relating to self-identity and habit [18,19,20].

Thirdly, and notwithstanding methodological limitations in the search strategy noted above, it is clear that research on individual environmental and health behaviors is weighted towards studies of travel behavior and physical activity/sedentary behavior. This focus reflects the environmental impacts of travel, a behavior which tends to be influenced by situational and financial factors; environmental concerns may not be a large motivational factor [161]. 

Almost all studies on public transport use and physical activity found significant positive associations, and a large proportion of studies on active travel also found associations in the same direction with physical activity. In contrast, the majority of studies on active travel and sedentary behaviors reported no evidence of significant associations between these behaviors. 

## 5. Conclusions

Our scoping review has confirmed that associations between active travel and physical activity have been extensively studied. There are also a range of studies analyzing active travel with sedentary behavior, and public transport use with physical activity.

Our scoping review has however identified some important areas which have received less attention from researchers and are recommended for future research. Travel-related behaviors and key health behaviors like smoking, alcohol intake and diet have been little studied to date. The broader sweep of environmental behaviors—including those related to food purchasing, food and household waste, recycling and energy use—and their associations with health behaviors also await detailed study. Longitudinal studies examining health and environmental behaviors are largely absent from the literature. Improving our understanding of causal pathways and changes in environmental and health behaviors over the life course should be a high priority for further research. This in turn would facilitate the integration of quantitative studies of behavior with the broader swathe of social scientific research on the psychosocial influences on everyday practices, including those relating to lifestyle types, self-identity and habit [18,19,20].

## Supplementary Files

Supplementary File 1
