# Supplementary material for: A Scoping Review of Observational Studies Examining Relationships between Environmental Behaviors and Health Behaviors
Source: Int J Environ Res Public Health. 2015 May 5;12(5):4833–58. doi: 10.3390/ijerph120504833 (PMC4454941; doi:10.3390/ijerph120504833)
Supplement: Supplementary File 1 [file ijerph-12-04833-s001.pdf]

Supplementary Information

A Scoping Review of Observational Studies Examining Relationships between Environmental Behaviors and Health Behaviors

---

Search strategy

Via OVID: PsychInfo (1967 to January Week 1 2014), (N = 1,547), Medline (1946 to November Week 3 2013), (N = 6,933), Medline in process (searched on 8 January, 2014), (N = 1,291), EconLit (1886 to December 2013 (N = 1875)

1. behavio#r$.mp.
2. (habit or habits).mp.
3. (behavior/ or habits/ or health behavior/ or risk reduction behavior/)
4. life style.mp.
5. lifestyle.mp.
6. 1 OR 2 OR 3 OR 4 OR 5
7. (ecological$ adj4 behavio#r$).mp.
8. (climate change adj4 behavio#r$).mp.
9. (pro-environmental$ adj4 behavio#r$).mp.
10. (proenvironmental$ adj4 behavio#r$).mp.
11. green$ behavio#r$.mp.
12. green$ consumerism.mp.
13. act environmentally.mp.
14. sustainable lifestyle.mp.
15. sustainable living.mp.
16. sustainable behavio#r$.mp.
17. climate change.mp.
18. global warming.mp.
19. greenhouse effect.mp.
20. carbon footprint.mp.
21. ecological footprint.mp.
22. (eco-friendly or environmental friendliness).mp.
23. climate change.mp.
24. global warming.mp.
25. greenhouse effect.mp.
26. environmental$ concern$.mp.
27. ecological$ concern$.mp.
28. environmental$ conscious.mp.
29. conservation of energy resources.mp.
30. conservation of natural resources.mp
31. environmental$ concern$.mp.
32. environmental impact$.mp.
33. sustainable agriculture.mp.
34. (environmental$ adj4 behavio#r$).mp.
35. 7 OR 8 OR 9 OR 10 OR 11 OR 12 OR 13 OR 14 OR 15 OR 16 OR 17 OR 18 OR 19 OR 20 OR 21 OR 22 OR 23 OR 24 OR 25 OR 26 OR 27 OR 28 OR 29 OR 30 OR 31 OR 32 OR 33 OR 34
36. 6 AND 35

37. food preferences.mp.
38. (food habits/ or food choice/)
39. (diet$ quality or healthy eating or diet$ patterns).mp.
40. smoking
41. smoker
42. smokers
43. smok$ adj15 tobacco
44. smok$ adj15 cigarett$
45. smok$ adj15 hookah
46. ((drug* adj3 abus*) or (drug* adj3 addict*) or (drug* adj3 depend*)).mp.
47. polydrug*.mp.
48. substance*.mp.
49. alcohol*.mp.
50. tranquil*.mp.
51. ((chemical* adj3 abus*) or (chemical* adj3 addict*) or (chemical* adj3 depend*)).mp.
52. narcotic*.mp.
53. opiate*.mp.
54. street drug*.mp.
55. solvent*.mp.
56. inhalant*.mp.
57. psychotropic*.mp.
58. intoxica*.mp.
59. 46 OR 47 OR 48 OR 49 OR 50 OR 51 OR 52 OR 53 OR 54 OR 55 OR 56 OR 57 OR 58
60. abus*.mp.
61. use*.mp.
62. misus*.mp.
63. usin*mp.
64. utiliz*.mp.
65. utilis*.mp.
66. depend*.mp.
67. addict*.mp.
68. illegal*.mp.
69. illicit*.mp.
70. habit*.mp.
71. withdraw*.mp.
72. behave*.mp.
73. abstinence*.mp.
74. abstain*.mp.
75. rehab*.mp.
76. intoxica*.mp.
77. non-prescri*.mp.
78. 60 OR 61 OR 62 OR 63 OR 64 OR 65 OR 66 OR 67 OR 68 OR 69 OR 70 OR 71 OR 72 OR 73 OR 74 OR 75 OR 76 OR 77
79. 59 AND 78
80. dual* adj diagnos*
81. exp substance-abuse/
82. exp drug-dependence /
83. exp alcohol-abuse /
84. exp alcoholism /
85. 37 OR 38 OR 39 OR 40 OR 41 OR 42 OR 43 OR 44 OR 45 OR 79 OR 80 OR 81 OR 82 OR 83 OR 84

86. exercise.tw.
87. physical activity.tw.
88. exertion.tw.
89. physical fitness.tw.
90. walking.tw.
91. (walk$ or cyclist$ or bicycl$ or bik$).mp.
92. 85 OR 86 OR 87 OR 88 OR 90 or 91
93. ((sav$ adj3 energy) or (sav$ adj3 water) or (sav$ adj3 electricity) or (energy adj3 efficien$) or energy conservation or energy consumption or low energy or low carbon or carbon footprint or carbon friendly or water conservation or water saving or wastewater reuse or household waste or domestic waste or household waste management or waste separation or reduc$ waste or reduc$ pack$ or organic food or organically grown or local food or locally grown or ecological consumption or food production practices or food related lifestyle or food waste or plate waste or food miles or public transport$ or (allotment adj10 vegetable$) or (allotment adj10 fruit) or (allotment adj10 bees)).mp.
94. recycling.ti,ab.
95. 93 or 94
96. (travel plan$ or transport plan$ or safe route$ or safer route$ or walking school bus$ or walking bus$ or ecological commut$ or ecological transport$ or mobility management plan$ or travel to work or commuter plan$ or travelsmart or walk to school).mp.
97. (Travel behaviour chang$ or travel behavior chang$).mp.
98. ((modal or mode) and (choice$ or distribution$ or selection$ or shift$ or split$ or substitut$ or switch$ or transfer$ or use$ or chang$ or modif$)).mp.
99. (travel$ or transportation$).tw. or transportation/
100. 98 AND 99
101. (Automobile$ or auto use$ or car or cars or mechani#ed transport$ or motori#ed transport$ or motorist$ or personal transport$ or road use$ or motor vehic$ or vkt$ or vmt$ or vehicle kilomet$ or vehicle mile$ or (driv$ adj5 (school$ or work$))).mp. or motor vehicles/
102. (walk$ or (cyclist$ or cycling or bicycl$ or bik$) or (bus or buses or busing or bussing or (train or trains or rail or railway) or public transport$) or (carshar$ or carpool$ or (car$ adj shar$) or (car$ adj pool$)) or (non-auto$ or non-motori#ed) or (telework$ or telecommut$)).mp. or walking/ or bicycling/
103. 101 and 102
104. (active commut$ or active travel$ or utilitarian walk$ or utilitarian cycl$ or green travel$ or greener travel$ or green transport$ or greener transport$ or ecological commut$ or ecological transport$ or ecotravel$ or ecotransport$ or ecocommut$).tw. or (active transport$.tw. not exp biological transport/ not exp carrier proteins/)
105. 103 or 104
106. 96 or 97 or 100 or 105
107. 95 or 36
108. 92 AND 107
109. 106 OR 108

BIOSIS via Web of Science 1969 to 03-01-2014, (N=10,302)

Limited to document types = article, taxa notes = humans language = English. Final set refined to OECD countries.

1. ((TI=(behavio*) OR TS=(behavio*) OR TI=(habit OR habits) OR TS=(habit OR habits) OR TI=(behavio* OR habits OR "health behavio*" OR "risk reduction behavio*") OR TS=(behavio* OR habits OR "health behavio*" OR "risk reduction behavio*") OR TI=(life style) OR TS=(life style) OR TI=(lifestyle) OR TS=(lifestyle)) NOT
TS=(" in vitro") NOT TI=("in vitro") NOT TS=(" in vivo") NOT TI=("in vivo") NOT TS=("organic acids") NOT TI=("organic acids") NOT TS=(membrane) NOT TI=(membrane) NOT TS=(cell) NOT

TI=(cell) NOT TS=(cells) NOT TI=(cells) NOT TS=(cellular) NOT TI=(cellular) NOT TS=(mice) NOT TI=(mice) NOT TS=(rats) NOT TI=(rats) NOT TS=(rat) NOT TI =(rat) NOT TS=(mouse) NOT TI=(mouse))

2. ((TI=(environmental* NEAR/4 behavio*) OR TI=(ecological NEAR/4 behavio*) OR TI=("climate change" NEAR/4 behavio*) OR TI=(pro-environmental* NEAR/4 behavio*) OR TI=(proenvironmental* NEAR/4 behavio*) OR TI=("green behavio*") OR TI=("green consumerism") OR TI=(" act environmentally") OR TI=("sustainable lifestyle") OR TI=("sustainable living") OR TI=("sustainable behavio*") OR TI=("climate change" OR "global warming" OR "greenhouse effect" OR "carbon footprint") OR TI=("ecological footprint") OR TI=(" eco-friendly" OR "environmental friendliness") OR TI=(" greenhouse effect") OR TI=("environmental* concern*") OR TI=("ecological* concern*") OR TI=(" environmental* conscious*") OR TI=("conservation of energy resources") OR TI=("conservation of natural resources") OR TI=("environmental impact*") OR TI=("sustainable agriculture") OR TI=(environmental* NEAR/4 behavio*) OR TS=(environmental* NEAR/4 behavio*) OR TS =(ecological NEAR/4 behavio*) OR TS =("climate change" NEAR/4 behavio*) OR TS =(pro-environmental* NEAR/4 behavio*) OR TS =(proenvironmental* NEAR/4 behavio*) OR TS =("green behavio*") OR TS =("green consumerism") OR TS =("act environmentally") OR TS =("sustainable lifestyle") OR TS =(" sustainable living") OR TS =("sustainable behavio*") OR TS=("climate change" OR "global warming" OR "greenhouse effect" OR "carbon footprint") OR TS=("ecological footprint") OR TS=("eco-friendly" OR "environmental friendliness") OR TS=("greenhouse effect") OR TS=("environmental* concern*") OR TS=("ecological* concern*") OR TS=("environmental* conscious*") OR TS=("conservation of energy resources") OR TS=("conservation of natural resources") OR TS=(" environmental impact*") OR TS=("sustainable agriculture") OR TI=(environmental* NEAR/4 behavio*)) NOT
TS=(" in vitro") NOT TI=("in vitro") NOT TS=(" in vivo") NOT TI=("in vivo") NOT TS=("organic acids") NOT TI=("organic acids") NOT TS=(membrane) NOT TI=(membrane) NOT TS=(cell) NOT TI=(cell) NOT TS=(cells) NOT TI=(cells) NOT TS=(cellular) NOT TI=(cellular) NOT TS=(mice) NOT TI=(mice) NOT TS=(rats) NOT TI=(rats) NOT TS=(rat) NOT TI =(rat) NOT TS=(mouse) NOT TI=(mouse))

3. #1 AND #2

4. ((TI=("food preferences") OR TI=("food habits" OR "food choice") OR TI=("diet* quality" OR "healthy eating" OR "diet* patterns") OR TI=(exercise) OR TI=(physical activity) OR TI=(exertion) OR TI=(physical fitness) OR TI=(walking) OR TI=(walk* or cyclist* or bicycl* or bik*) OR TI=(smoking) OR TI=(smoker) OR TI=(smokers) OR TI=(smok* NEAR/15 tobacco) OR TI=(smok* NEAR/15 cigarett*) OR TI=(smok* NEAR/15 hookah) OR TI=(dual* NEAR/2 diagnos*) OR TI=(substance-abuse) OR TI=(drug-dependence) OR TI=(alcohol-abuse) OR TI=(alcoholism) OR TS=("food preferences") ORTS=("food habits" OR "food choice") OR TS=("diet* quality" OR "healthy eating" OR "diet* patterns") OR TS=(exercise) OR TS=("physical activity") OR TS=(exertion) OR TS=("physical fitness") OR TS=(walking) OR TS=(walk* or cyclist* or bicycl* or bik*) OR TS=(smoking) OR TS=(smoker) OR TS=(smokers) OR TS=(smok* NEAR/15 tobacco) OR TS=(smok* NEAR/15 cigarett*) OR TS=(smok* NEAR/15 hookah) OR TS=(dual* NEAR/2 diagnos*) OR TS=(substance-abuse) OR TS=(drug-dependence) OR TS=(alcohol-abuse) OR TS=(alcoholism)) NOT
TS=(" in vitro") NOT TI=("in vitro") NOT TS=(" in vivo") NOT TI=("in vivo") NOT TS=("organic acids") NOT TI=("organic acids") NOT TS=(membrane) NOT TI=(membrane) NOT TS=(cell) NOT TI=(cell) NOT TS=(cells) NOT TI=(cells) NOT TS=(cellular) NOT TI=(cellular) NOT TS=(mice) NOT TI=(mice) NOT TS=(rats) NOT TI=(rats) NOT TS=(rat) NOT TI =(rat) NOT TS=(mouse) NOT TI=(mouse))

5. ((TI=((drug* NEAR/3 abus*) or (drug* NEAR/3 addict*) or (drug* NEAR/3 depend*)) OR TI=(polydrug*) OR TI= (substance*) OR TI=(alcohol*) OR TI=(tranquil*) OR TI=((chemical* NEAR/3 abus*) or (chemical* NEAR/3 addict*) or (chemical* NEAR/3 depend*)) OR TI=(narcotic*) OR TI=(opiat*) OR TI=(street drug*) OR TI=(solvent*) OR TI=(inhalant*) OR TI=(psychotropic*)

OR TI=(intoxica*) OR TS= ((drug* NEAR/3 abus*) or (drug* NEAR/3 addict*) or (drug* NEAR/3 depend*)) OR TS = (polydrug*) OR TS = (substance*) OR TS =(alcohol*) OR TS =(tranquil*) OR TS =((chemical* NEAR/3 abus*) or (chemical* NEAR/3 addict*) or (chemical* NEAR/3 depend*)) OR TS =(narcotic*) OR TS =(opiat*) OR TS =(street drug*) OR TS =(solvent*) OR TS =(inhalant*) ORTS =(psychotropic*)) NOT
TS=(" in vitro") NOT TI=("in vitro") NOT TS=(" in vivo") NOT TI=("in vivo") NOT TS=("organic acids") NOT TI=("organic acids") NOT TS=(membrane) NOT TI=(membrane) NOT TS=(cell) NOT TI=(cell) NOT TS=(cells) NOT TI=(cells) NOT TS=(cellular) NOT TI=(cellular) NOT TS=(mice) NOT TI=(mice) NOT TS=(rats) NOT TI=(rats) NOT TS=(rat) NOT TI =(rat) NOT TS=(mouse) NOT TI=(mouse))

6. ((TI=(abus*) OR TI=(use*) OR TI=(misus*) OR TI=(usin*) OR TI=(utiliz*) OR TI=(utilis*) OR TI=(depend*) OR TI=(addict*) OR TI=(illegal*) OR TI=(illicit*) OR TI=(habit*) OR TI=(withdraw*) OR TI=(behavi*) OR TI=(abstinence*) OR TI=(abstain*) OR TI=(rehab*) OR TI=(intoxica*) OR TI=(non-prescri*) OR TS=(abus*) OR TS =(use*) OR TS =(misus*) OR TS =(usin*) OR TS =(utiliz*) OR TS =(utilis*) OR TS =(depend*) OR TS =(addict*) OR TS =(illegal*) OR TS =(illicit*) OR TS =(habit*) OR TS =(withdraw*) OR TS =(behavi*) OR TS =(abstinence*) OR TS =(abstain*) OR TS =(rehab*) OR TS =(intoxica*) OR TS =(non-prescri*)) NOT
TS=(" in vitro") NOT TI=("in vitro") NOT TS=(" in vivo") NOT TI=("in vivo") NOT TS=("organic acids") NOT TI=("organic acids") NOT TS=(membrane) NOT TI=(membrane) NOT TS=(cell) NOT TI=(cell) NOT TS=(cells) NOT TI=(cells) NOT TS=(cellular) NOT TI=(cellular) NOT TS=(mice) NOT TI=(mice) NOT TS=(rats) NOT TI=(rats) NOT TS=(rat) NOT TI =(rat) NOT TS=(mouse) NOT TI=(mouse))
7. #5 AND #6
8. #4 or #7
9. ((TI=(sav* NEAR/3 energy) or TI=(sav* NEAR/3 water) or TI=(sav* NEAR/3 electricity) or TI=(energy NEAR/3 efficien*) or TI=("energy conservation") or TI=("energy consumption") or TI=("low energy") or TI=("low carbon") or TI=("carbon footprint") or TI=("carbon friendly") or TI=("water conservation") or TI=("water saving") or TI=("wastewater reuse") or TI=("household waste") or TI=("domestic waste") or TI=("household waste management") or TI=("waste separation") or TI=("reduc* waste") or TI=("reduc* pack*") or TI=("organic food") or TI=("organically grown") or TI=("local food") or TI=("locally grown") or TI=("ecological consumption") or TI=("food production practices") or TI=("food related lifestyle") or TI=("food waste") or TI=("plate waste") or TI=("food miles") or TI=("public transport*") or TI=(allotment NEAR/10 vegetable*) or TI=(allotment NEAR/10 fruit) or TI=(allotment NEAR/10 bees) orTI=(recycling) or TS=(sav* NEAR/3 energy) or TS =(sav* NEAR/3 water) or TS =(sav* NEAR/3 electricity) or TS =(energy NEAR/3 efficien*) or TS =("energy conservation") or TS =("energy consumption") or TS =("low energy") or TS =("low carbon") or TS =("carbon footprint") or TS =("carbon friendly") or TS =("water conservation") or TS =("water saving") or TS =("wastewater reuse") or TS =("household waste") or TS =("domestic waste") or TS =("household waste management") or TS =("waste separation") or TS =("reduc* waste") or TS =("reduc* pack*") or TS =("organic food") or TS =("organically grown") orTS =("local food") or TS =("locally grown") or TS =("ecological consumption") or TS =("food production practices") or TS =("food related lifestyle") or TS =("food waste") or TS =("plate waste") or TS =("food miles") or TS =("public transport*") or TS =(allotment NEAR/10 vegetable*) or TS =(allotment NEAR/10 fruit) or TS =(allotment NEAR/10 bees) orTS =(recycling)) NOT
TS=(" in vitro") NOT TI=("in vitro") NOT TS=(" in vivo") NOT TI=("in vivo") NOT TS=("organic acids") NOT TI=("organic acids") NOT TS=(membrane) NOT TI=(membrane) NOT TS=(cell) NOT TI=(cell) NOT TS=(cells) NOT TI=(cells) NOT TS=(cellular) NOT TI=(cellular) NOT TS=(mice) NOT TI=(mice) NOT TS=(rats) NOT TI=(rats) NOT TS=(rat) NOT TI =(rat) NOT TS=(mouse) NOT TI=(mouse))

10. ((TI= (“travel plan*”) or TI= (“transport plan*”) or TI= (“safe route*”) or TI= (“safer route*”) or TI= (“walking school bus*”) or TI= (“walking bus*”) or TI= (“ecological commut*”) or TI= (“ecological transport*”) or TI= (“mobility management plan*”) or TI= (“travel to work”)or TI= (“commuter plan*”) or TI= (travelsmart) or TI= (“walk to school”) or TI= (“travel behaviour chang*”) or TI=(“travel behavior chang*”) OR TS= (“travel plan*”) or TS = (“transport plan*”) or TS = (“safe route*”) or TS = (“safer route*”) or TS = (“walking school bus*”) or TS = (“walking bus*”) or TS = (ecological commut*) or TS = (“ecological transport*”) or TS = (“mobility management plan*”) or TS = (“travel to work”) or TS = (“commuter plan*”) or TS = (travelsmart) or TS = (“ walk to school”) orTS = (“travel behaviour chang*”) or TS =(“travel behavior chang*”)) NOT TS=(“ in vitro”) NOT TI=(“in vitro”) NOT TS=(“ in vivo”) NOT TI=(“in vivo”) NOT TS=(“organic acids”) NOT TI=(“organic acids”) NOT TS=(membrane) NOT TI=(membrane) NOT TS=(cell) NOT TI=(cell) NOT TS=(cells) NOT TI=(cells) NOT TS=(cellular) NOT TI=(cellular) NOT TS=(mice) NOT TI=(mice) NOT TS=(rats) NOT TI=(rats) NOT TS=(rat) NOT TI =(rat) NOT TS=(mouse) NOT TI=(mouse))
11. ((TI= ((modal or mode) and (choice* or distribution* or selection* or shift* or split* or substitut* or switch* or transfer* or use* or chang* or modif*)) OR
TS= ((modal or mode) and (choice* or distribution* or selection* or shift* or split* or substitut* or switch* or transfer* or use* or chang* or modif*))) NOT
TS=(“ in vitro”) NOT TI=(“in vitro”) NOT TS=(“ in vivo”) NOT TI=(“in vivo”) NOT TS=(“organic acids”) NOT TI=(“organic acids”) NOT TS=(membrane) NOT TI=(membrane) NOT TS=(cell) NOT TI=(cell) NOT TS=(cells) NOT TI=(cells) NOT TS=(cellular) NOT TI=(cellular) NOT TS=(mice) NOT TI=(mice) NOT TS=(rats) NOT TI=(rats) NOT TS=(rat) NOT TI =(rat) NOT TS=(mouse) NOT TI=(mouse))
12. ((TI= (travel*) or
TI=(transportation*) OR
TS= (travel*) or
TS=(transportation*)) NOT
TS=(“ in vitro”) NOT TI=(“in vitro”) NOT TS=(“ in vivo”) NOT TI=(“in vivo”) NOT TS=(“organic acids”) NOT TI=(“organic acids”) NOT TS=(membrane) NOT TI=(membrane) NOT TS=(cell) NOT TI=(cell) NOT TS=(cells) NOT TI=(cells) NOT TS=(cellular) NOT TI=(cellular) NOT TS=(mice) NOT TI=(mice) NOT TS=(rats) NOT TI=(rats) NOT TS=(rat) NOT TI =(rat) NOT TS=(mouse) NOT TI=(mouse))
13. #11 AND #12
14. ((TI=(Automobile* or “auto use*” or car or cars or “mechani* transport*” or “motori* transport*” or motorist* or “personal transport*” or “road use*” or “motor vehic*” or vkt* or vmt* or “vehicle kilomet*” or “vehicle mile*” or (driv* NEAR/5 (school* or work*))) OR
TS=(Automobile* or “auto use*” or car or cars or “mechani* transport*” or “motori* transport*” or motorist* or “personal transport*” or “road use*” or “motor vehic*” or vkt* or vmt* or “vehicle kilomet*” or “vehicle mile*” or (driv* NEAR/5 (school* or work*)))) NOT
TS=(“ in vitro”) NOT TI=(“in vitro”) NOT TS=(“ in vivo”) NOT TI=(“in vivo”) NOT TS=(“organic acids”) NOT TI=(“organic acids”) NOT TS=(membrane) NOT TI=(membrane) NOT TS=(cell) NOT TI=(cell) NOT TS=(cells) NOT TI=(cells) NOT TS=(cellular) NOT TI=(cellular) NOT TS=(mice) NOT TI=(mice) NOT TS=(rats) NOT TI=(rats) NOT TS=(rat) NOT TI =(rat) NOT TS=(mouse) NOT TI=(mouse))
15. ((TI=(walk* or (cyclist* or cycling or bicycl* or bik*) or (bus or buses or busing or bussing or (train or trains or rail or railway) or “public transport*”) or (carshar* or carpool* or (car* NEAR/3 shar*) or (car* NEAR/3 pool*)) or (“non-auto*” or “non-motori*”) or (telework* or telecommut*)) OR
TS=(walk* or (cyclist* or cycling or bicycl* or bik*) or (bus or buses or busing or bussing or (train or trains or rail or railway) or “public transport*”) or (carshar* or carpool* or (car* NEAR/3 shar*) or (car* NEAR/3 pool*)) or (“non-auto*” or “non-motori*”) or (telework* or telecommut*))) NOT

TS=(“ in vitro”) NOT TI=(“in vitro”) NOT TS=(“ in vivo”) NOT TI=(“in vivo”) NOT TS=(“organic acids”) NOT TI=(“organic acids”) NOT TS=(membrane) NOT TI=(membrane) NOT TS=(cell) NOT TI=(cell) NOT TS=(cells) NOT TI=(cells) NOT TS=(cellular) NOT TI=(cellular) NOT TS=(mice) NOT TI=(mice) NOT TS=(rats) NOT TI=(rats) NOT TS=(rat) NOT TI =(rat) NOT TS=(mouse) NOT TI=(mouse))
16. #14 AND #15
17. ((TI=(“active commut*” or “active travel*” or “utilitarian walk*” or “utilitarian cycl*” or “green travel*” or “greener travel*” or “green transport*” or “greener transport*” or “ecological commut*” or “ecological transport*” or ecotravel* or ecotransport*or ecocommut*) OR
TI= (“active transport*”) OR
TS=(“active commut*” or “active travel*” or “utilitarian walk*” or “utilitarian cycl*” or “green travel*” or “greener travel*” or “green transport*” or “greener transport*” or “ecological commut*” or “ecological transport*” or ecotravel* or ecotransport*or ecocommut*) OR
TS=(active transport*)) NOT
TS=(“ in vitro”) NOT TI=(“in vitro”) NOT TS=(“ in vivo”) NOT TI=(“in vivo”) NOT TS=(“organic acids”) NOT TI=(“organic acids”) NOT TS=(membrane) NOT TI=(membrane) NOT TS=(cell) NOT TI=(cell) NOT TS=(cells) NOT TI=(cells) NOT TS=(cellular) NOT TI=(cellular) NOT TS=(mice) NOT TI=(mice) NOT TS=(rats) NOT TI=(rats) NOT TS=(rat) NOT TI =(rat) NOT TS=(mouse) NOT TI=(mouse))
18. #16 or #17
19. #10 OR #13 OR #18
20. #3 or #9
21. #8 AND #20
22. #19 OR #21

Social Science Citation Index via Web of Science, 1956 to 08-01-2014 (N=7,589)

Limited to document types = article. Final set refined to OECD countries.
1. (TI=(behavio*) OR TS=(behavio*) OR TI=(habit OR habits) OR TS=(habit OR habits) OR TI=(behavio* OR habits OR “health behavio*” OR “risk reduction behavio*”) OR TS=(behavio* OR habits OR “health behavio*” OR “risk reduction behavio*”) OR TI=(life style) OR TS=(life style) OR TI=(lifestyle) OR TS=(lifestyle))
2. (TI=(environmental* NEAR/4 behavio*) OR TI=(ecological NEAR/4 behavio*) OR TI=(“climate change” NEAR/4 behavio*) OR TI=(pro-environmental* NEAR/4 behavio*) OR TI=(proenvironmental* NEAR/4 behavio*) OR TI=(“green behavio*”) OR TI=(“green consumerism”) OR TI=(“ act environmentally”) OR TI=(“sustainable lifestyle”) OR TI=(“sustainable living”) OR TI=(“sustainable behavio*”) OR TI=(“climate change” OR “global warming” OR “greenhouse effect” OR “carbon footprint”) OR TI=(“ecological footprint”) OR TI=(“ eco-friendly” OR “environmental friendliness”) OR TI=(“ greenhouse effect”) OR TI=(“environmental* concern*”) OR TI=(“ecological* concern*”) OR TI=(“ environmental* conscious*”) OR TI=(“conservation of energy resources”) OR TI=(“conservation of natural resources”) OR TI=(“environmental impact*”) OR TI=(“sustainable agriculture”) OR TI=(environmental* NEAR/4 behavio*) OR TS=(environmental* NEAR/4 behavio*) OR TS =(ecological NEAR/4 behavio*) OR TS =(“climate change” NEAR/4 behavio*) OR TS =(pro-environmental* NEAR/4 behavio*) OR TS =(proenvironmental* NEAR/4 behavio*) OR TS =(“green behavio*”) OR TS =(“green consumerism”) OR TS =(“act environmentally”) OR TS =(“sustainable lifestyle”) OR TS =(“ sustainable living”) OR TS =(“sustainable behavio*”) OR TS=(“climate change” OR “global warming” OR “greenhouse effect” OR “carbon footprint”) OR TS=(“ecological footprint”) OR TS=(“eco-friendly” OR “environmental friendliness”) OR TS=(“greenhouse effect”) OR TS=(“environmental* concern*”) OR TS=(“ecological* concern*”) OR TS=(“environmental* conscious*”) OR TS=(“conservation of

energy resources”) OR TS=(“conservation of natural resources”) OR TS=(“ environmental impact*”) OR TS=(“sustainable agriculture”) OR TI=(environmental* NEAR/4 behavio*))
3. #1 AND #2
4. (TI=(“food preferences”) OR TI=(“food habits” OR “food choice”) OR TI=(“diet* quality” OR “healthy eating” OR “diet* patterns”) OR TI=(exercise) OR TI=(physical activity) OR TI=(exertion) OR TI=(physical fitness) OR TI=(walking) OR TI=(walk* or cyclist* or bicycl* or bik*) OR TI=(smoking) OR TI=(smoker) OR TI=(smokers) OR TI=(smok* NEAR/15 tobacco) OR TI=(smok* NEAR/15 cigarett*) OR TI=(smok* NEAR/15 hookah) OR TI=(dual* NEAR/2 diagnos*) OR TI=(substance-abuse) OR TI=(drug-dependence) OR TI=(alcohol-abuse) OR TI=(alcoholism) OR TS=(“food preferences”) OR TS=(“food habits” OR “food choice”) OR TS=(“diet* quality” OR “healthy eating” OR “diet* patterns”) OR TS=(exercise) OR TS=(“physical activity”) OR TS=(exertion) OR TS=(“physical fitness”) OR TS=(walking) OR TS=(walk* or cyclist* or bicycl* or bik*) OR TS=(smoking) OR TS=(smoker) OR TS=(smokers) OR TS=(smok* NEAR/15 tobacco) OR
TS=(smok* NEAR/15 cigarett*) OR TS=(smok* NEAR/15 hookah) OR TS=(dual* NEAR/2 diagnos*) OR TS=(substance-abuse) OR TS=(drug-dependence) OR TS=(alcohol-abuse) OR TS=(alcoholism))
5. (TI=((drug* NEAR/3 abus*) or (drug* NEAR/3 addict*) or (drug* NEAR/3 depend*)) OR TI=(polydrug*) OR TI= (substance*) OR TI=(alcohol*) OR TI=(tranquil*) OR TI=((chemical* NEAR/3 abus*) or (chemical* NEAR/3 addict*) or (chemical* NEAR/3 depend*)) OR TI=(narcotic*) OR TI=(opiat*) OR TI=(street drug*) OR TI=(solvent*) OR TI=(inhalant*) OR TI=(psychotropic*) OR TI=(intoxica*) OR TS= ((drug* NEAR/3 abus*) or (drug* NEAR/3 addict*) or (drug* NEAR/3 depend*)) OR TS = (polydrug*) OR TS = (substance*) OR TS =(alcohol*) OR TS =(tranquil*) OR TS =((chemical* NEAR/3 abus*) or (chemical* NEAR/3 addict*) or (chemical* NEAR/3 depend*)) OR TS =(narcotic*) OR TS =(opiat*) OR TS =(street drug*) OR TS =(solvent*) OR TS =(inhalant*) OR TS =(psychotropic*))
6. (TI=(abus*) OR TI=(use*) OR TI=(misus*) OR TI=(usin*) OR TI=(utiliz*) OR TI=(utilis*) OR TI=(depend*) OR TI=(addict*) OR TI=(illegal*) OR TI=(illicit*) OR TI=(habit*) OR TI=(withdraw*) OR TI=(behavi*) OR TI=(abstinence*) OR TI=(abstain*) OR TI=(rehab*) OR TI=(intoxica*) OR TI=(non-prescri*) OR TS=(abus*) OR TS =(use*) OR TS =(misus*) OR TS =(usin*) OR TS =(utiliz*) OR TS =(utilis*) OR TS =(depend*) OR TS =(addict*) OR TS =(illegal*) OR TS =(illicit*) OR TS =(habit*) OR TS =(withdraw*) OR TS =(behavi*) OR TS =(abstinence*) OR TS =(abstain*) OR TS =(rehab*) OR TS =(intoxica*) OR TS =(non-prescri*))
7. #5 AND #6
8. #4 or #7
9. (TI=(sav* NEAR/3 energy) or TI=(sav* NEAR/3 water) or TI=(sav* NEAR/3 electricity) or TI=(energy NEAR/3 efficien*) or TI=(“energy conservation”) or TI=(“energy consumption”) or TI=(“low energy”) or TI=(“low carbon”) or TI=(“carbon footprint”) or TI=(“carbon friendly”) or TI=(“water conservation”) or TI=(“water saving”) or TI=(“wastewater reuse”) or TI=(“household waste”) or TI=(“domestic waste”) or TI=(“household waste management”) or TI=(“waste separation”) or TI=(“reduc* waste”) or TI=(“reduc* pack*”) or TI=(“organic food”) or TI=(“organically grown”) or TI=(“local food”) or TI=(“locally grown”) or TI=(“ecological consumption”) or TI=(“food production practices”) or TI=(“food related lifestyle”) or TI=(“food waste”) or TI=(“plate waste”) or TI=(“food miles”) or TI=(“public transport*”) or TI=(allotment NEAR/10 vegetable*) or TI=(allotment NEAR/10 fruit) or TI=(allotment NEAR/10 bees) or TI=(recycling) or TS=(sav* NEAR/3 energy) or TS =(sav* NEAR/3 water) or TS =(sav* NEAR/3 electricity) or TS =(energy NEAR/3 efficien*) or TS =(“energy conservation”) or TS =(“energy consumption”) or TS =(“low energy”) or TS =(“low carbon”) or TS =(“carbon footprint”) or TS =(“carbon friendly”) or TS =(“water conservation”) or TS =(“water saving”) or TS =(“wastewater reuse”) or TS =(“household waste”) or TS =(“domestic waste”) or TS =(“household waste

management”) or TS =(“waste separation”) or TS =(“reduc* waste”) or TS =(“reduc* pack*”) or TS =(“organic food”) or
TS =(“organically grown”) orTS =(“local food”) or TS =(“locally grown”) or TS =(“ecological consumption”) or TS =(“food production practices”) or TS =(“food related lifestyle”) or TS =(“food waste”) or TS =(“plate waste”) or TS =(“food miles”) or TS =(“public transport*”) or TS =(allotment NEAR/10 vegetable*) or TS =(allotment NEAR/10 fruit) or TS =(allotment NEAR/10 bees) orTS =(recycling))
10. (TI= (“travel plan*”) or TI= (“transport plan*”) or TI= (“safe route*”) or TI= (“safer route*”) or TI= (“walking school bus*”) or TI= (“walking bus*”) or TI= (“ecological commut*”) or TI= (“ecological transport*”) or TI= (“mobility management plan*”) or TI= (“travel to work”)or TI= (“commuter plan*”) or TI= (travelsmart) or TI= (“walk to school”) or TI= (“travel behaviour chang*”) or TI=(“travel behavior chang*”) OR TS= (“travel plan*”) or TS = (“transport plan*”) or TS = (“safe route*”) or TS = (“safer route*”) or TS = (“walking school bus*”) or TS = (“walking bus*”) or TS = (ecological commut*) or TS = (“ecological transport*”) or TS = (“mobility management plan*”) or TS = (“travel to work”) or TS = (“commuter plan*”) or TS = (travelsmart) or TS = (“ walk to school”) or TS = (“travel behaviour chang*”) or TS =(“travel behavior chang*”))
11. (TI= ((modal or mode) and (choice* or distribution* or selection* or shift* or split* or substitut* or switch* or transfer* or use* or chang* or modif*)) OR
TS= ((modal or mode) and (choice* or distribution* or selection* or shift* or split* or substitut* or switch* or transfer* or use* or chang* or modif*)))
12. (TI= (travel*) or
TI=(transportation*) OR
TS= (travel*) or
TS=(transportation*))
13. #11 AND #12
14. (TI=(Automobile* or “auto use*” or car or cars or “mechani* transport*” or “motori* transport*” or motorist* or “personal transport*” or “road use*” or “motor vehic*” or vkt* or vmt* or “vehicle kilomet*” or “vehicle mile*” or (driv* NEAR/5 (school* or work*))) OR
TS=(Automobile* or “auto use*” or car or cars or “mechani* transport*” or “motori* transport*” or motorist* or “personal transport*” or “road use*” or “motor vehic*” or vkt* or vmt* or “vehicle kilomet*” or “vehicle mile*” or (driv* NEAR/5 (school* or work*))))
15. (TI=(walk* or (cyclist* or cycling or bicycl* or bik*) or (bus or buses or busing or bussing or (train or trains or rail or railway) or “public transport*”) or (carshar* or carpool* or (car* NEAR/3 shar*) or (car* NEAR/3 pool*)) or (“non-auto*” or “non-motori*”) or (telework* or telecommut*)) OR
TS=(walk* or (cyclist* or cycling or bicycl* or bik*) or (bus or buses or busing or bussing or (train or trains or rail or railway) or “public transport*”) or (carshar* or carpool* or (car* NEAR/3 shar*) or (car* NEAR/3 pool*)) or (“non-auto*” or “non-motori*”) or (telework* or telecommut*)))
16. #14 AND #15
17. (TI=(“active commut*” or “active travel*” or “utilitarian walk*” or “utilitarian cycl*” or “green travel*” or “greener travel*” or “green transport*” or “greener transport*” or “ecological commut*” or “ecological transport*” or ecotravel* or ecotransport*or ecocommut*) OR
TI= (“active transport*”) OR
TS=(“active commut*” or “active travel*” or “utilitarian walk*” or “utilitarian cycl*” or “green travel*” or “greener travel*” or “green transport*” or “greener transport*” or “ecological commut*” or “ecological transport*” or ecotravel* or ecotransport*or ecocommut*) OR
TS= (active transport*))
18. #16 or #17
19. #10 OR #13 OR #18
20. #3 or #9
21. #8 AND #20
22. #19 OR #21

Table S1. Description of included studies in the scoping review of bivariate analyses of health and environmental behaviors.

ID/1st Author/ Date	Country National/Regional	Survey/Recruitment /Date of Collection	Analysis	Number Analysed	Sample Age	Data Collection Method (of Behaviors Analysed Together) [a]	Environmental Behavior/s	Health Behavior/s	Category/s for this Review
**Active Travel and Physical Activity**									
[26] Abbott 2009	Australia Regional	2006 Healthy Kids Queensland (HKQ)	Cross-sectional	2076	Children 6–16	*Travel:* questionnaire *Physical activity:* pedometer	Active travel to school	Physical activity-steps	AT/PA
[27] Alexander 2005	UK Regional	2004 Four schools	Cross-sectional	92	13–14	*Transport*: questionnaire *Physical activity:* accelerometer	Active travel to school	Moderate to vigorous physical activity	AT/PA
[28] Anderson 2009	Denmark Regional	1983 Danish Youth and Sports Study	Cross-sectional	1249	15–19	Questionnaire	Active travel to school	Leisure time physical activity	AT/PA
[29] Anderson 2011	Denmark Regional	1997–1998 and 2003–04 European Youth Heart Study	Cross-sectional & Longitudinal	589 baseline& 334 follow-up	Children 9 & 15	*Transport*: questionnaire *Physical activity:* accelerometer	Cycling to school compared to non-cyclists Plus change in above	Total physical activity	AT/PA
[30] Baig 2009	UK Regional	(Date NR) Three middle schools	Cross-sectional	673	Children 11–14	Questionnaire	Active travel to school	Physical activity during school time	AT/PA

Table S1. *Cont.*

ID/1st Author/ Date	Country National/Regional	Survey/Recruitment /Date of Collection	Analysis	Number Analysed	Sample Age	Data Collection Method (of Behaviors Analysed Together) [a]	Environmental Behavior/s	Health Behavior/s	Category/s for this Review
[31] Borrestad 2013	Norway Regional	2009 Two schools	Cross-sectional	58	Children 10–12	Questionnaire	Active travel to school	Moderate to vigorous physical activity	AT/PA
[32] Brophy 2011	UK Nationally representative	2000–2002 Millennium Cohort Study	Cross-sectional	13,641	Children	Parental interviews using computer assisted questionnaire	Walking to school	Sedentary behavior<br>Organised and non-organised physical activity	AT/SB<br>AT/PA
[33] Butler 2007	Canada Nationally representative	2003 Canadian Community Health Survey (CCHS)	Cross-sectional	77,953	Adults 15+	Self-completed computer-assisted telephone interview	Active travel	Sedentary occupation<br>Smoking<br>Leisure time physical activity	AT/SB<br>AT/OHB<br>AT/PA
[34] Carse 2013	UK Regional	2009 Commuting and Health in Cambridge study	Cross-sectional	1164 (3784 trips)	Adults 16+	Self- completed questionnaire	Cycling for transport	Walking for leisure	AT/PA

Table S1. *Cont.*

ID/1st Author/ Date	Country National/Regional	Survey/Recruitment /Date of Collection	Analysis	Number Analysed	Sample Age	Data Collection Method (of Behaviors Analysed Together) [a]	Environmental Behavior/s	Health Behavior/s	Category/s for this Review
[35] Carver 2011	Australia Regional	2001–2006 Children Living in Active Neighbourhoods	Cross-sectional at 3 time points	1214, 607, 443	Children 5–17	*Transport:* questionnaire (parent's of younger children completed) *Physical activity:* accelerometer	Active travel to school and other destinations	Moderate to vigorous physical activity	AT/PA
[36] Cerin 2009	Australia Regional	2003–2004 High and low walkable suburbs of Adelaide	Cross-sectional	2194	Adults 20–60	Questionnaire	Active travel	Leisure time physical activity	AT/PA
[37] Chillon 2011	10 European Cities	2006–8 HELENA (Healthy Lifestyle in Europe by Nutrition in Adolescence)	Cross-sectional	3112	Children 12–17	*Transport*: questionnaire *Physical activity:* accelerometer and questionnaire	Active travel	Total physical activity Moderate to vigorous physical activity Non-travel physical activity	AT/PA
[38] Chillon 2010	Estonia/ Sweden Regional	1998/99 European Youth Heart Study	Cross-sectional	2271	Children 9–16	*Transport*: questionnaire *Physical activity:* activity monitor	Active travel to school	Moderate to vigorous physical activity	AT/PA

Table S1. *Cont.*

ID/1st Author/ Date	Country National/Regional	Survey/Recruitment /Date of Collection	Analysis	Number Analysed	Sample Age	Data Collection Method (of Behaviors Analysed Together) [a]	Environmental Behavior/s	Health Behavior/s	Category/s for this Review
[39] Cooper 2005	Denmark Regional	1997/98 European Youth Heart Study	Cross-sectional	323	Children 9	*Transport*: questionnaire *Physical activity:* accelerometer	Active travel to school	Moderate to vigorous physical activity	AT/PA
[40] Cooper 2003	UK Regional	2002 Five urban primary schools	Cross-sectional	114	Children 10	*Transport:* questionnaire *Physical activity:* accelerometer	Walking to school	Moderate to vigorous physical activity Meeting physical activity guidelines	AT/PA
[41] Cooper 2012	UK Regional	2006 & 2009 Personal and Environmental Associations with Children's Health (PEACH) project	Cross-sectional & Longitudinal	500	Children 11–12	*Transport*: questionnaire *Physical activity:* accelerometer	Active travel to school Plus change in above	Moderate to vigorous physical activity	AT/PA
[42] Cooper 2010	UK Regional	2006 Personal and Environmental Associations with Children's Health (pilot study)	Cross-sectional	137	Children 10–11	*Transport:* questionnaire *Physical activity:* accelerometer	Active travel to school	Moderate to vigorous physical activity	AT/PA
[43] Cooper 2008	Denmark Regional	1997 & 2003 European Youth Heart Study	Cross-sectional at 2 time points	384	Children 8–10	*Transport:* questionnaire *Physical activity:* accelerometer	Active travel to school	Moderate to vigorous physical activity	AT/PA

Table S1. *Cont.*

ID/1st Author/ Date	Country National/Regional	Survey/Recruitment /Date of Collection	Analysis	Number Analysed	Sample Age	Data Collection Method (of Behaviors Analysed Together) [a]	Environmental Behavior/s	Health Behavior/s	Category/s for this Review
[44] Cooper 2006	Denmark Regional	1998-9 European Youth Heart Study	Cross-sectional	531	Children 9 & 15	*Transport:* questionnaire *Physical activity:* accelerometer	Active travel to school	Total physical activity Moderate to vigorous physical activity	AT/PA
[45] Daly-Smith 2011	UK Regional	2006 64 schools	Cross-sectional	5422	Children 8–9	Questionnaire	Active travel to school	Meeting physical activity guidelines	AT/PA
[46] Davis 2011	UK Regional	2007–2008 Random sample of patients from GP lists	Cross-sectional	214	Adults 70+	*Transport:* self-completed log *Physical activity:* accelerometer	Public transport use Active travel	Physical activity – steps Moderate to vigorous physical activity	PT/PA AT/PA
[47] de Bruijn 2009	Holland Regional	2006 Amsterdam Growth And Health Longitudinal Study (AGAHLS)	Cross-sectional	317	Adults Mean 42	Questionnaire	Active travel	Leisure-time physical activity (walking and cycling)	AT/PA
[48] De Cocker 2007	Belgium Nationally representative	2005 Random sample from public record office	Cross-sectional	1239	Adults 25–75	*Transport:* telephone interview questionnaire *Physical activity:* pedometer	Active travel	Total physical activity	AT/PA

Table S1. *Cont.*

ID/1st Author/ Date	Country National/Regional	Survey/Recruitment /Date of Collection	Analysis	Number Analysed	Sample Age	Data Collection Method (of Behaviors Analysed Together) [a]	Environmental Behavior/s	Health Behavior/s	Category/s for this Review
[49] Dollman 2007	Australia Regional	2005 Schools in Southern Australia	Cross-sectional	1643	Children 9–15	Parent completed questionnaire	Active travel to school	Leisure-time physical activity Neighbourhood active travel	AT/PA
[50] Dombois 2007	Switzerland Regional	2004 Three alpine communities	Cross-sectional	901	Adults 18+	Telephone interview	Active travel to work or to leisure activities	Meeting physical activity guidelines	AT/PA
[51] Drygas 2009	Poland Nationally representative	2002-2005 National Polish Health Survey (WOBASZ Project)	Cross-sectional	12,552	Adults 20–74	Interview administered questionnaire	Active travel to work	Leisure-time physical activity Occupational physical activity	AT/PA
[52] Ducheyne 2012	Belgium Regional	2010-2011 Random sample of schools	Cross-sectional	850	Children 10–12	Parent- completed questionnaire	Cycling to school	Sedentary behavior Organised physical activity	AT/SB AT/PA
[53] Duncan 2008	New Zealand Regional	2004-2005 39 schools	Cross-sectional	1513	Children 5–16	*Transport:* Self or parent completed questionnaire *Physical activity:* pedometer	Active travel to school	Total physical activity	AT/PA

Table S1. *Cont.*

ID/1st Author/ Date	Country National/Regional	Survey/Recruitment /Date of Collection	Analysis	Number Analysed	Sample Age	Data Collection Method (of Behaviors Analysed Together) [a]	Environmental Behavior/s	Health Behavior/s	Category/s for this Review
[54] Engbers 2010	Netherlands National	2008 Random sample of employed persons	Cross-sectional	799	Mixed 13-65	Self-completed internet survey	Cycling to work	Sedentary behavior<br>Sedentary occupation<br>Cycling for leisure<br>Organised physical activity<br>Meeting physical activity guidelines	AT/SB<br>AT/PA
[55] Faulkner 2013	Canada Regional	2010-2011 16 schools	Cross-sectional	785	Children 10–12	*Transport:* Parent completed questionnaire<br>*Physical activity:* accelerometer	Walking to school	Moderate to vigorous physical activity	AT/PA
[56] Farrer 2013	New Zealand Nationally representative	2008/2009 National Survey of Children and Young People's Physical Activity and Dietary Behaviors	Cross-sectional	679	Children 10–16	*Transport, sedentary behaviors and diet*: interviews<br>*Physical activity:* accelerometer	Active travel	Moderate to vigorous physical activity<br>Sedentary behavior<br>Fruit and vegetable intake	AT/PA<br>AT/OHB

Table S1. *Cont.*

ID/1st Author/ Date	Country National/Regional	Survey/Recruitment /Date of Collection	Analysis	Number Analysed	Sample Age	Data Collection Method (of Behaviors Analysed Together) [a]	Environmental Behavior/s	Health Behavior/s	Category/s for this Review
[57] Ford 2007	UK Regional	(year NR) Two schools	Cross-sectional	239	Children 5–11	*Commuting mode:* Parent- completed questionnaire *Energy intake:* Parent and child completed questionnaire *Physical activity*: accelerometer	Active travel to school	Energy intake Total physical activity	AT/OHB AT/PA
[58] Freeman 2013	USA Regional	2003 New York City Community Health Survey (CHS)	Cross-sectional	9802	Adults 18+	Telephone survey	Active travel over 10 blocks	Physical activity	AT/PA
[59] Fulton 2005	USA Nationally representative	1996	Cross-sectional	1395	Children 9–18	Telephone survey	Active travel to school	Physical activity frequency	AT/PA
[60] Furie 2012	USA Nationally representative	2006-2007 & 2009-2010 National Health and Nutrition Examination Survey (NHANES)	Cross-sectional	9933	Adults ≥20	NR	Active travel	Smoking Leisure time and occupational physical activity	AT/OHB AT/PA

Table S1. *Cont.*

ID/1st Author/ Date	Country National/Regional	Survey/Recruitment /Date of Collection	Analysis	Number Analysed	Sample Age	Data Collection Method (of Behaviors Analysed Together) [a]	Environmental Behavior/s	Health Behavior/s	Category/s for this Review
[61] Goodman 2011	UK Regional	2002-2006 Convenience sample	Cross-sectional	345	Children 8–13	*Transport:* questionnaire *Physical activity:* accelerometer	Active travel to school Active travel to other destinations	Moderate to vigorous physical activity (not in active travel)	AT/PA
[62] Gordon-Larsen 2005	USA Nationally representative	2001-2002 The National Longitudinal Study of Adolescent Health (Add Health)	Cross-sectional	10771	Adults 18–28	Questionnaire	Active travel to work or school	Meeting physical activity guidelines	AT/PA
[63] Goodman 2012	UK Regional	2010 iConnect study	Cross-sectional	3463	Adults 18–96	Questionnaire	Active travel for any purpose	Leisure time physical activity	AT/PA
[64] Grow 2008	US Regional	(Date NR) Three metropolitan areas	Cross-sectional	211	Children 5–18	Parent or adolescent completed questionnaire	Active travel to recreation sites	Leisure time physical activity	AT/PA
[65] Harten 2004	Australia Regional	(Date NR) 11 schools	Cross-sectional	136	Children 11–12	*Transport:* interview *Physical activity:* computerised activity diary	Active travel to any destination	Total physical activity Energy expenditure	AT/PA
[66] Hearst 2013	USA Regional	2006-2008 TREC–IDEA and ECHO studies	Cross-sectional	550	Adults	Questionnaire	Walking for transport	Walking for leisure	AT/PA

Table S1. *Cont.*

ID/1st Author/ Date	Country National/Regional	Survey/Recruitment /Date of Collection	Analysis	Number Analysed	Sample Age	Data Collection Method (of Behaviors Analysed Together) [a]	Environmental Behavior/s	Health Behavior/s	Category/s for this Review
[67] Heelan 2005	USA Regional	(year NR) Five rural schools in a state	Cross-sectional	320	Children 9–11	Self-completed questionnaires	Active travel to school	Sedentary behavior<br>Total physical activity	AT/SB<br>AT/PA
[68] Hohepa 2008	New Zealand Regional	2005 Three schools in Auckland	Cross-sectional	236	Children 12–18	*Travel:* questionnaire<br>*Physical activity:* pedometer	Walking to school	Physical activity steps	AT/PA
[69] Johnson 2010	USA Regional	2006-7 Four urban schools	Cross-sectional	547	Children 10–14	*Travel:* questionnaire<br>*Physical activity:* pedometer	Active travel to school	Physical activity steps	AT/PA
[70] King 2011	England Regional	2006-7 Gateshead Millennium Study	Cross-sectional	480	Children 7+	*Active travel, parental variables*: Parent- completed questionnaire<br>*Physical and sedentary activity*: Actigraph	Active travel to school	Child's sedentary behavior<br>Parental TV / screen time<br>Parental use of active transport<br>Child's total and moderate to vigorous physical activity	AT/SB<br>AT/PA
[71] Kwasniewska 2010	Poland Nationally representative	2002-2005 National Multicentre Health Survey WOBASZ	Cross-sectional	7,280	Adults 20–74	Interviewer-administered questionnaire	Active travel to work	Smoking<br>Leisure time physical activity<br>Occupational physical activity	AT/OHB<br>AT/PA

Table S1. *Cont.*

ID/1st Author/ Date	Country National/Regional	Survey/Recruitment /Date of Collection	Analysis	Number Analysed	Sample Age	Data Collection Method (of Behaviors Analysed Together) [a]	Environmental Behavior/s	Health Behavior/s	Category/s for this Review
[72] Kwasniewska 2010	Poland Nationally representative	2002-2005 National Multicentre Health Survey WOBASZ	Cross-sectional	6401	Adults 20–74	Interviewer-administered questionnaire	Active travel to work	Smoking<br>Alcohol<br>Calorie intake	AT/OHB<br>AT/PA
[73] Lachapelle 2012	USA Regional	2009 Random-digit phone survey	Cross-sectional	530	Adults 18+	Telephone interviews	Public transport use<br>Walking to work	Walking frequency to transport node or to work	PT/PA<br>AT/PA
[74] Landsberg 2008	Germany Regional	2004-2005 Kiel Obesity Prevention Study	Cross-sectional	626	Children 14	Self-completed questionnaire supervised at school	Active travel to school	Alcohol (habitual)<br>Smoking<br>Dietary pattern<br>Sedentary behavior<br>Total physical activity<br>Meeting physical activity guidelines	AT/SB<br>AT/OHB<br>AT/PA
[75] Larouche 2011	Canada Regional	2009-10 Canadian Assessment of Physical Literacy (CAPL)	Cross-sectional	315	Children 9–12	*Travel:* questionnaire<br>*Physical activity:* pedometer	Active travel to school	Physical activity - steps	AT/PA

Table S1. *Cont.*

ID/1st Author/ Date	Country National/Regional	Survey/Recruitment /Date of Collection	Analysis	Number Analysed	Sample Age	Data Collection Method (of Behaviors Analysed Together) [a]	Environmental Behavior/s	Health Behavior/s	Category/s for this Review
[76] Leslie 2010	Australia Regional	2006 231 Schools in three states	Cross-sectional	2961	Children 10–14	Questionnaire	Active travel to school	Meeting physical activity guidelines Physical activity outside school time	AT/PA
[77] Loucaides 2007	Canada Regional	2000 Four urban and four rural schools	Cross-sectional	2688	Children Mean 15	Questionnaire	Active travel to school	Moderate to vigorous physical activity	AT/PA
[78] Marques 2013	Portugal Regional	2010/2011 SALTA Project	Cross-sectional	636	Children 11	*Transport:* questionnaire *Physical activity:* accelerometer	Active travel from school	Moderate to vigorous physical activity	AT/PA
[79] Martin 2007	USA Nationally representative	2004 Youth Media Campaign Longitudinal Survey (YMCLS)	Cross-sectional	7,433	Children 9–15 years	*Active commuting:* parent reported *Physical and Sedentary behavior*: child reported	Active travel to school	Sedentary behavior Organised physical activity Leisure time physical activity	AT/SB AT/PA
[80] Mendoza 2011	USA	2003–2004 NHANES	Cross-sectional	789	12–19	*Transport:* questionnaire *Physical activity:* accelerometer	Active travel to school	Moderate to vigorous physical activity	AT/PA

Table S1. *Cont.*

ID/1st Author/ Date	Country National/Regional	Survey/Recruitment /Date of Collection	Analysis	Number Analysed	Sample Age	Data Collection Method (of Behaviors Analysed Together) [a]	Environmental Behavior/s	Health Behavior/s	Category/s for this Review
[81] Merom 2008	Australia Regional	2003 Randomly selected from major metropolitan areas	pre/post intervention	1100	Adults 18–65	Telephone survey	Active travel to work (single day and usual commute)	Total physical activity	AT/PA
[82] Metcalf 2004	UK Regional	(Date NR) Early Bird study	Cross-sectional	275	Children 5	*Travel:* questionnaire *Physical activity:* accelerometer	Walking to school	Total physical activity	AT/PA
[83] Molina-García 2010	Spain Regional	2009 Two universities	Cross-sectional	518	Students Mean 22	Questionnaire	Active travel to the university	Total physical activity	AT/PA
[84] Morckel 2014	USA Multi-regional	(date NR) Random selection of university employees	Cross-sectional	99	Adults 18+	Self-reported on-line survey	Public transport use<br>Active travel	Leisure time and occupation physical activity	PT/PA<br>AT/PA
[85] Mota 2010	Portugal Regional	2006 11 urban schools	Cross-sectional	1121	Children 13–17	Questionnaire	Active travel from school	Physical activities outside school time	AT/PA
[86] Murtagh 2011	Ireland Regional	2009 Four urban schools	Cross-sectional	140	Children 9–11	*Travel:* questionnaire *Physical activity:* pedometer	Active travel from school	Physical activity - steps	AT/PA
[87] Naumann 2009	USA	(Date NR)	Cross-sectional	406	Adults Over 64	Telephone survey	Walking for short journeys	Walking for exercise or leisure	AT/PA

Table S1. *Cont.*

ID/1st Author/ Date	Country National/Regional	Survey/Recruitment /Date of Collection	Analysis	Number Analysed	Sample Age	Data Collection Method (of Behaviors Analysed Together) [a]	Environmental Behavior/s	Health Behavior/s	Category/s for this Review
[88] Nilsson 2009	Norway, Estonia & Portugal Multi-national	1997-2000 European Youth Heart Study	Cross-sectional	1,327	Children, 9 and 15 years	*Active travel and TV viewing*: Self-completed questionnaire *Sedentary time and physical activity*: pedometer	Active travel to school	Sedentary behavior Moderate to vigorous physical activity	AT/SB AT/PA
[89] Oreskovic 2009	USA Regional	2007-8 Asthmatic children	Cross-sectional	176	Children 5–15	Parent completed questionnaire	Active travel to school	Frequency of physical activity	AT/PA
[90] Østergaard 2012	Denmark Regional	2009 33 schools	Cross-sectional	3847	Children 12–16	Questionnaire	Active travel to school	Leisure time physical activity	AT/PA
[91] Østergaard 2013	Norway Nationally representative	2005-6 Physical Activity among Norwegian Children Study	Cross-sectional	1694	Children 9 and 15	Researcher completed questionnaires	Active travel to school	Leisure time physical activity	AT/PA
[92] Owen 2012	England Multi-regional	2006-2007 Child Heart and Heath Study in England (CHASE)	Cross-sectional	2,035	Children 9–10 years	*Travel:* Self-completed questionnaire *Activity/sedentary*: Actigraph	Active travel to school Public transport use	Sedentary behavior Total physical activity Moderate to vigorous physical activity	PT/PA PT/SB AT/SB AT/PA

Table S1. *Cont.*

ID/1st Author/ Date	Country National/Regional	Survey/Recruitment /Date of Collection	Analysis	Number Analysed	Sample Age	Data Collection Method (of Behaviors Analysed Together) [a]	Environmental Behavior/s	Health Behavior/s	Category/s for this Review
[93] Pabayo 2012	Canada Regional	2009 Raising healthy Eating and Active Living Kids in Alberta (REAL Kids Alberta)	Cross-sectional	688	Children 10–11	*Travel:* parent completed questionnaire *Physical activity:* pedometer	Active travel to school	Physical activity - steps<br>Meeting physical activity guidelines	AT/PA
[94] Panter 2011	UK Regional	2007-8 SPEEDY study	Cross-sectional	1824	Children 9–10	*Transport:* questionnaire *Physical activity:* accelerometer	Active travel to school	Total physical activity<br>Moderate to vigorous physical activity	AT/PA
[95] Rissel 2013	Australia Regional	2012 University staff and students	Cross-sectional	3737	Adults	Online survey	Public transport use<br>Active travel	Meeting physical activity guidelines	PT/PA<br>AT/PA
[96] Robertson-Wilson 2008	Canada Regional	2005–2006 School Health Action, Planning and Evaluation System (SHAPES)–Ontario study	Cross-sectional	21,345	Children 14–18	Self-completed questionnaire in class	Active travel to school	Smoking<br>Sedentary behavior<br>Low, moderate or vigorous physical activity	AT/SB<br>AT/OHB<br>AT/PA

Table S1. *Cont.*

ID/1st Author/ Date	Country National/Regional	Survey/Recruitment /Date of Collection	Analysis	Number Analysed	Sample Age	Data Collection Method (of Behaviors Analysed Together) [a]	Environmental Behavior/s	Health Behavior/s	Category/s for this Review
[97] Rosenberg 2006	US Regional	1990–2 Sports, Play, and Active Recreation for Kids	Cross-sectional	2007	Children 9–10	*Transport:* questionnaire *Physical activity:* accelerometer	Walking, cycling skateboarding to school	Total physical activity	AT/PA
[98] Roth 2012	UK Nationally representative	2008 Health Survey for England	Cross-sectional	4468 (603 wore accelerometer)	Children 5–15	*Transport:* questionnaire *Physical activity:* accelerometer and questionnaire	Active travel to school	Other Active travel, Moderate to vigorous physical activity Meeting physical activity guidelines	AT/PA
[99] Sahlqvist 2013	UK Regional	2010–2011 iConnect study	Longitudinal	1628	18+	Questionnaire	Change in active travel	Total physical activity, Total leisure time physical activity	AT/PA
[100] Sahlqvist 2012	UK Regional	2010 iConnect study	Cross-sectional	3339	18+	Questionnaire	Active travel	Total physical activity Leisure time physical activity	AT/PA
[101] Sahlqvist 2012	Australia Regional	2009 Queensland	Cross-sectional	1813	Adults	Questionnaire	Cycling for transport	Meeting physical activity guidelines	AT/PA

Table S1. *Cont.*

ID/1st Author/ Date	Country National/Regional	Survey/Recruitment /Date of Collection	Analysis	Number Analysed	Sample Age	Data Collection Method (of Behaviors Analysed Together) [a]	Environmental Behavior/s	Health Behavior/s	Category/s for this Review
[102] Saksvig 2012	US Regional	2003-5 Trial of Activity for Adolescent Girls (TAAG)	Cross-Sectional & Longitudinal	3076 (cross.) and 1017 (long.)	Children 11–14	*Transport:* questionnaire *Physical activity:* accelerometer	Walking for transport before and after school	Moderate to vigorous physical activity before and after school Total physical activity Sedentary time	AT/PA AT/SB
[103] Santos 2009	Portugal Regional	2006 Six urban schools	Cross-sectional	721	Children 13–18	Questionnaire	Active travel to school	Organised physical activity outside of school time Non-organised physical activity outside of school time	AT/PA
[104] Sayers 2012	USA Regional	2007 Three schools in Columbia, MO	Cross-sectional	77	Children 5–11	*Transport:* questionnaire *Physical activity:* accelerometer	Walking school bus	Moderate to vigorous physical activity	AT/PA
[105] Schofield 2005	New Zealand Regional	(Date NR) Worksites in Auckland and Christchurch	Cross-sectional	181	Adult	*Transport:* questionnaire *Physical activity:* pedometer	Frequency of active travel	Occupational physical activity	AT/PA

Table S1. *Cont.*

ID/1st Author/ Date	Country National/Regional	Survey/Recruitment /Date of Collection	Analysis	Number Analysed	Sample Age	Data Collection Method (of Behaviors Analysed Together) [a]	Environmental Behavior/s	Health Behavior/s	Category/s for this Review
[106] Sirard 2005	USA Regional	(Date NR) Four urban and four suburban schools	Cross-sectional	219	Children 9–11	*Transport:* questionnaire *Physical activity:* accelerometer	Active travel to school	Total physical activity Moderate to vigorous physical activity	AT/PA
[107] Sisson 2008	USA Regional	2005–2006 University students	Cross-sectional	50	~21	*Transport:* travel log *Physical activity:* accelerometer and travel log	Cycling for transport	Physical activity - steps Moderate to vigorous physical activity	AT/PA
[108] Slingerland 2012	Netherlands Regional	2009–2010 Three schools	Cross-sectional	73	Children 15	*Transport:* activity log *Physical activity:* accelerometer	Active travel to school and leisure	Total physical activity (energy expenditure)	AT/PA
[109] Smith 2012	UK Regional	2007–2008 SPEEDY study	Longitudinal	812	Children 9–10	*Transport:* questionnaire *Physical activity:* accelerometer	Active travel to school	Moderate to vigorous physical activity	AT/PA
[110] Smith 2012	UK Regional	2007–2008 SPEEDY study	Cross-sectional	1859	Children 9–10	*Transport:* questionnaire *Physical activity:* accelerometer	Active travel to school Active travel to other destinations	Moderate to vigorous physical activity	AT/PA

Table S1. *Cont.*

ID/1st Author/ Date	Country National/Regional	Survey/Recruitment /Date of Collection	Analysis	Number Analysed	Sample Age	Data Collection Method (of Behaviors Analysed Together) [a]	Environmental Behavior/s	Health Behavior/s	Category/s for this Review
[111] Spinks 2006	Australia Regional	2001 & 2003 Childhood Injury Prevention Study (CHIPS)	Cross-sectional	518	Children 5–12	*Transport:* interview *Physical activity:* diary	Active travel to school	Meeting physical activity guidelines	AT/PA
[112] Sugiyama 2010	Australia Regional	2003-2004 Random sample of households in study area	Cross-sectional	1524	Adults 20–65	Self-completed questionnaire	Active travel	Sedentary behavior<br>Leisure time physical activity<br>Occupational physical activity	AT/SB<br>AT/PA
[113] van Sluijs 2009	UK Regional	2002–2004 Avon Longitudinal Study of Parents and Children	Cross-sectional	4688	Children 11	*Transport:* carer completed questionnaire *Physical activity:* accelerometer	Active travel to school	Total physical activity	AT/PA
[114] Voss 2010	UK Regional	2007-8 East of England Healthy Hearts study	Cross-sectional	6085	Children 10–15	Questionnaire	Active travel to school	Total physical activity	AT/PA
[115] Wen 2010	Australia Regional	2006 Central Sydney Walk to School Research Program	Cross-sectional	1362	Children 10–13	Parent-completed questionnaire	Active travel to school	Sedentary behavior<br>Physical activity	AT/SB<br>AT/PA

Table S1. *Cont.*

ID/1st Author/ Date	Country National/Regional	Survey/Recruitment /Date of Collection	Analysis	Number Analysed	Sample Age	Data Collection Method (of Behaviors Analysed Together) [a]	Environmental Behavior/s	Health Behavior/s	Category/s for this Review
[116] Yang 2012	UK Regional	2009 Travel and Health in Cambridge	Cross-sectional	475	16+	*Transport:* questionnaire *Physical activity:* accelerometer	Active travel to work	Moderate to vigorous physical activity	AT/PA
[117] Yelavich 2008	New Zealand Regional	2004 39 schools	Cross-sectional	1006	Children 5–11	Parent completed questionnaire	Walking to school	Total physical activity	AT/PA
**Active Travel and Sedentary Behavior or other Health Behaviors not Included Above**									
[118] Chau 2012	Australia Nationally representative	2007, 2008 Australian National Health Survey	Cross-sectional	10,785	15–69	Interviews using computer assisted questionnaire	Active travel	Occupational sedentary activity	AT/SB
[119] Dunton 2009	USA Nationally representative	2006 American Time Use Survey	Cross-sectional	10,984	21+	Interviewers using computer assisted telephone interview	Active travel	Sedentary behavior	AT/SB
[120] Granzin 1991	USA Regional	(year NR)	Cross-sectional	340	Adults	Self-completed survey in interviewer presence	Recycling Active travel	Sedentary behavior	AT/SB Rec/HB
[121] Mota 2007	Portugal Regional	2004 from 11 public schools	Cross-sectional	705	Children 12–17 years	Self-completed questionnaire in class	Active travel	Sedentary behavior	AT/SB

Table S1. *Cont.*

ID/1st Author/ Date	Country National/Regional	Survey/Recruitment /Date of Collection	Analysis	Number Analysed	Sample Age	Data Collection Method (of Behaviors Analysed Together) [a]	Environmental Behavior/s	Health Behavior/s	Category/s for this Review
[122] Wong 2011	Canada Regional	2009 Ontario Student Drug Use and Health Survey (OSDUHS)	Cross-sectional	3633	Children 12–18	Self-completed questionnaire in class	Active travel to school	Smoking<br>Drinking alcohol<br>Sedentary behavior<br>Moderate to vigorous physical activity	AT/SB<br>AT/OHB
[123] Wang 2010	Finland Multi-regional	1972-2002 Every five years population surveys	Cross-sectional	58,208	Adults 25–74	Self-completed questionnaire	Active travel to work	Alcohol use<br>Smoking	AT/OHB
**Public Transport and Physical Activity Not Included Above**									
[124] Besser 2005	USA Sub-sample of nationally representative survey	2001 National Household Travel Survey (NHTS)	Cross-sectional	3312 transit users	Adults 18+	Telephone interview	Public transport use	Walking to transit node	PT/PA
[125] Cerin 2007	Australia Regional	(year NR) Physical Activity in Localities and Community Environments (PLACE) study	Cross-sectional	2650	Adults 20–65	Self- completed questionnaire	Public transport use (assumed from frequency of walking to public transport node)	Walking for transport (mins)	PT/PA

Table S1. *Cont.*

ID/1st Author/ Date	Country National/Regional	Survey/Recruitment /Date of Collection	Analysis	Number Analysed	Sample Age	Data Collection Method (of Behaviors Analysed Together) [a]	Environmental Behavior/s	Health Behavior/s	Category/s for this Review
[126] Dolnicar 2010	Switzerland Nationally representative	2004 Panel of residents	Cross-sectional (year long study)	3050 (4,471 trips)	Adults	Self- completed questionnaire and logs	Travel mode to tourist destination	Active pursuits on the trip Bicycling for transport on the trip	PT/PA
[127] Edwards 2008	USA Nationally representative	2001 National Household Travel Survey	Cross-sectional	28,711	Adults 18+	Travel diaries reported at interview	Public transport use	Walking time	PT/PA
[128] Goetzke 2011	Germany Multi-regional	2002 Mobility in Germany (MiG)	Cross-sectional	3,821	15+	NR	Public transport use	Active travel to school/work	PT/PA
[129] Lachapelle 2011	USA Multi-regional	2003 The Neighborhood Quality of Life Study (NQLS)	Cross-sectional	1,237	Adults 20–65	*Transit use:* Interviewer-administered questionnaire *Physical activity*: Accelerometer	Public transport use	Moderate physical activity Meeting physical activity guidelines	PT/PA
[130] Lachapelle 2009	USA Regional	2001-2002 Strategies for Metropolitan Atlanta's Regional Transportation and Air Quality (SMARTRAQ)	Cross-sectional	4156	Adults 16–70	*Travel mode*: 2-day travel diary via computer assisted telephone call *Physical activity:* GIS	Public transport use	Moderate physical activity Meeting physical activity guidelines	PT/PA

Table S1. *Cont.*

ID/1st Author/ Date	Country National/Regional	Survey/Recruitment /Date of Collection	Analysis	Number Analysed	Sample Age	Data Collection Method (of Behaviors Analysed Together) [a]	Environmental Behavior/s	Health Behavior/s	Category/s for this Review
[131] Lee 2007	USA Regional	2002 Walkable and Bikable Communities	Cross-sectional	438	Adults	Telephone Survey	Public transport use	Active travel	PT/PA
[132] MacDonald 2010	USA Regional	2006–2007 and 2008–2009 Sample addresses within mile of proposed light rail transit line	Before and after	660	Adults	Telephone interview	Public transport use	Walking activity Vigorous physical activity	PT/PA
[133] Moudon 2005	USA Regional	2002 Walkable and Bikable Communities (WBC) project	Cross-sectional	608	Adults 18+	Telephone interview	Public transport use	Cycling	PT/PA
[134] Villanueva 2008	Australia Regional	2006 Convenience sample of University students	Cross-sectional	103	Adults 16+	Self-completed questionnaire and activity log *Step counts*: Pedometer	Public transport use	Physical activity – steps Leisure time physical activity	PT/PA
[135] Wasfi	Canada Regional	2003 Origin-Destination (OD) Survey	Cross-sectional	6913	Adults 18+	Telephone interview	Public transport mode	Walking	PT/PA

Table S1. *Cont.*

ID/1st Author/ Date	Country National/Regional	Survey/Recruitment /Date of Collection	Analysis	Number Analysed	Sample Age	Data Collection Method (of Behaviors Analysed Together) [a]	Environmental Behavior/s	Health Behavior/s	Category/s for this Review
[136] Wener 2007	USA Regional	(date NR) Convenience sample of regular commuters	Cross-sectional	177	Adults	Self-completed questionnaire *Step counts*: Pedometer	Public transport use	Physical activity Steps Minutes walked	PT/PA
[137] Fuller 2013	USA Regional	2006 Philadelphia Neighborhood Food Environment Study	Cross-sectional	1266	Adults	Self-completed questionnaire	Transport mode	Fruit and vegetable consumption	PT/OHB
**Car Use and Physical Activity Not Included Above**									
[138] Lemstra 2013	Canada Regional	2010 Tribal community survey	Cross-sectional	204	Children 10–16	Parental-completed questionnaire	Car transport by parent	Meeting physical activity guidelines	CU/PA
[139] Swanson 2012	Canada Regional	2007 Random sample from telephone book	Cross-sectional	1026	Adults 18+	*Physical activity*: telephone interview *Driving time*: self-completed questionnaire	Weekly driving time	Meeting physical activity guidelines	CU/PA
[140] Wen 2006	Australia Regional	2003 The New South Wales (NSW) Continuous Health Survey	Cross-sectional	6810	Adults 16+	Telephone interview	Car use	Meeting physical activity guidelines	CU/PA

Table S1. *Cont.*

ID/1st Author/ Date	Country National/Regional	Survey/Recruitment /Date of Collection	Analysis	Number Analysed	Sample Age	Data Collection Method (of Behaviors Analysed Together) [a]	Environmental Behavior/s	Health Behavior/s	Category/s for this Review
**Car Use and Other Health Behaviors**									
[141] Wennburg 2010	Sweden Regional	1985–1999 Vasterbotten Intervention Program	Cross-sectional analysis in a prospective nested case-control study	531	Adults 30–60	Self-completed questionnaire	Car use	Smoking	CU/OHB
**Organic Food**									
[142] Kesse-Guyot 2013	France National	2009–2011 Nutrinet-Santé cohort	Cross-sectional	54,311	Adults 18+	Internet survey	Organic food consumption	Dietary composition<br>Physical activity<br>Smoking	OF
[143] Magnusson 2003	Sweden National	1998 Random sample	Cross-sectional	1154	Adults 18–65	Self-completed questionnaire	Environmentally friendly behavior	Frequency of organic food purchase	OF
[144] Miao 2013	USA Regional	(date NR) Random selection of university employees	Cross-sectional	1185	Adults 21–83 years	Self-completed questionnaire	Purchase refillable products<br>Purchase bio-degradable products	Purchase organic food	OF

Table S1. *Cont.*

ID/1st Author/ Date	Country National/Regional	Survey/Recruitment /Date of Collection	Analysis	Number Analysed	Sample Age	Data Collection Method (of Behaviors Analysed Together) [a]	Environmental Behavior/s	Health Behavior/s	Category/s for this Review
[145] Nie 2011	USA National	2003 A food consumer survey	Cross-sectional	956	Adults 18+	NR	Shopping at farmers markets	Buying organic<br>Cooking<br>Following a special diet	OF<br>LF
[146] Petersen 2013	Denmark National	1996–2002 Danish National Birth Cohort	Cross-sectional	60,773	Pregnant women	Food and other data from a mailed food frequency questionnaire, other data from telephone interviews	Organic food use	Smoking<br>Alcohol<br>Eating meat<br>Vegetarian / vegan<br>Physical activity<br>Eating specific foods<br>Nutrient intake<br>Dietary pattern	OF
[147] Thogersen 2006	Denmark National	1998–2000 Random sample of consumers	Cross-sectional	1100	Adults 18+	Telephone interview	Public transport use<br>Active travel<br>Recycling	Buy organic food	OF
[148] Torjusen 2010	Norway National	2002–2007 Norwegian Mother and Child Cohort Study (MoBa)	Cross-sectional	63,561	Pregnant women	Self-completed questionnaires	Organic food use	Vegetarianism<br>Physical activity<br>Smoking<br>Alcohol use	OF

Table S1. *Cont.*

ID/1st Author/ Date	Country National/Regional	Survey/Recruitment /Date of Collection	Analysis	Number Analysed	Sample Age	Data Collection Method (of Behaviors Analysed Together) [a]	Environmental Behavior/s	Health Behavior/s	Category/s for this Review
[149] Torjusen 2012	Norway National	2002-2007 Norwegian Mother and Child Cohort Study (MoBa)	Cross-sectional	63,808	Pregnant women	Self-completed questionnaires	Organic food use	Vegetarianism Dietary components Energy intake	OF
**Locally Sourced Food Not Included Above**									
[150] Burgess 2009	Australia Regional	2005 Aboriginal Indigenous residents in a remote community recruited via an outreach preventive health checks program	Cross-sectional	298	Adults 15–54	Interviewer-administered questionnaire	Caring for the community composite score	Smoking Drinking alcohol Exercise frequency Consumption of store and bush food	LF
[151] Litt 2011	USA Regional	2006-2007 random sample households and community gardens	Cross-sectional	436	Adults 18+	Interview	Gardening participation	Fruit and vegetable consumption	LF

Table S1. *Cont.*

ID/1st Author/ Date	Country National/Regional	Survey/Recruitment /Date of Collection	Analysis	Number Analysed	Sample Age	Data Collection Method (of Behaviors Analysed Together) [a]	Environmental Behavior/s	Health Behavior/s	Category/s for this Review
[152] MacMillan Uribe 2012	USA Regional	2009 Convenience sample of CSA members	Retrospective before and after	115	Adults 18+	Online survey	Previous Community Supported Agriculture membership	Fruit and vegetable intake	LF
[153] Maples 2013	USA Multi-regional	2012 Sample of urban individuals responsible for household shopping	Cross-sectional	1023	Adults 18+	Online survey	Buying directly from producers	Walking activity level	LF
**Plate Waste and Health Behaviors**									
[155] Baik 2009	South Korea Regional	2008–2009 Five elementary schools in a rural province	Cross-sectional	407 parent reported 91 observed	Children 6–9	*Plate waste*: Parent completed questionnaire; weighed observation at school (sub-sample) *Health behaviors*: Parent completed questionnaire *Step count*: 5-day Pedometer Assessed for adherence to National Guidelines	Plate waste	Dietary composition Meeting physical activity guidelines	PW/HB

Table S1. *Cont.*

ID/1st Author/ Date	Country National/Regional	Survey/Recruitment /Date of Collection	Analysis	Number Analysed	Sample Age	Data Collection Method (of Behaviors Analysed Together) [a]	Environmental Behavior/s	Health Behavior/s	Category/s for this Review
[156] Marlette 2005	USA Regional	2002 From three middle schools	Cross-sectional	743	Children 11–12 years	Weighing plate waste, observing competitive food item purchase	Plate waste	Competitive food item purchase e.g. unhealthy snacks	PW/HB

[a] Self reported unless otherwise stated; NR not reported; GIS Geographic Information Systems; CSA community supported agriculture; Key to categories for this review: AT/SB Active transportation (walking or cycling) and sedentary behaviour; AT/OHB Active transportation (walking or cycling) and other health behaviour (smoking, alcohol use, diet); PT/PA Public transportation and physical activity; PT/OHB Public transportation and other health behaviour (smoking, alcohol use, diet); CU/PA Car use and physical activity; CU/OHB Car use and other health behaviour (smoking, alcohol use, diet); OF Organic food and any other environmental or health behaviour; LF Local food and any other environmental or health behaviour; PW/HB Plate waste and any health behaviour; Rec/HB Recycling and any health behaviour.
The full references for these papers can be found in the main article

Table S2. Description of systematic reviews included in the scoping review of bivariate analyses of health and environmental behaviors in observational studies.

ID/1st Author/Date	Inclusion Criteria	Number of Studies in Reviews Meeting Our Search Criteria (Analysing Relationship between Health and Environmental Behavior(s) in OECD Counties)	Environmental Behavior/s	Health Behavior/s	Notes
[8] Faulkner 2009	All study designs<br>English language<br>Children 5–18<br>Active travel as exposure variable<br>Objective measures of physical activity and/or body weight	11	Active travel	Physical activity	13 studies found in total, all examined associations between active travel and physical activity. Two studies from non-OECD countries.
[9] Lee 2008	All study designs<br>Children (any age below university)<br>Active commuting to school as exposure<br>Physical activity or body weight as outcome(s)	23	Active travel	Physical activity	32 studies were identified, 25 examined active commuting and physical activity, 18 examined active commuting and weight status. Two AT/PA studies from non-OECD countries.
[10] Schoeppe 2013	Observational studies<br>English language<br>Children 3-18<br>Active travel or independent mobility as exposure(s)<br>Physical activity, sedentary behavior or weight status as outcome(s)	44	Active travel	Sedentary behavior<br>Physical activity	52 studies found in total, 42 examined active travel and physical activity and five examined active travel and sedentary behavior. Three AT/PA studies from non-OECD countries.

Table S2. *Cont.*

ID/1st Author/Date	Inclusion Criteria	Number of Studies in Reviews Meeting Our Search Criteria (Analysing Relationship between Health and Environmental Behavior(s) in OECD Counties)	Environmental Behavior/s	Health Behavior/s	Notes
[11] Wanner 2012	All study designs<br>Published in English, German or French<br>Adults<br>Published up to October 2010<br>Active transportation as exposure variable<br>Physical activity or body weight as outcome(s)<br>Published in peer-reviewed journals	14	Active travel	Physical activity	46 articles based on 36 unique studies found which examined active travel and physical activity (17 articles) and active travel and body weight (38 articles). Three AT/PA articles from non-OECD countries.
[12] Rissel 2012	All study designs<br>Published 2002–2012<br>Adults	8	Public transport	Physical activity	18 articles were included in an appendix that examined other aspects e.g. public transport and BMI, ease of access to public transport and physical activity
[154] Kamphuis 2006	Observational studies<br>English language<br>Published 1980–2004<br>Population based<br>Age 18–60 years<br>Established market economy<br>Fruit and vegetable intake as outcome variable	4	Locally grown food<br>Vegetable garden/home-grown produce	Fruit and vegetable intake	24 studies found in total which examined factors external to the individual that determined fruit and vegetable intake

The full references for these papers can be found in the main article

Referenses

26. Abbott, R.A.; Macdonald, D.; Nambiar, S.; Davies, P.S.W. The association between walking to school, daily step counts and meeting step targets in 5- to 17-year-old Australian children. *Pediatr. Exerc. Sci.* **2009**, *21*, 520–532.
27. Alexander, L.M.; Inchley, J.; Todd, J.; Currie, D.; Cooper, A.R.; Currie, C. The broader impact of walking to school among adolescents: Seven day accelerometry based study. *BMJ Br. Med. J.* **2005**, *331*, 1061–1062.
28. Andersen, L.B.; Lawlor, D.A.; Cooper, A.R.; Froberg, K.; Anderssen, S.A. Physical fitness in relation to transport to school in adolescents: The Danish youth and sports study. *Scand. J. Med. Sci. Sports* **2009**, *19*, 406–411.
29. Andersen, L.B.; Wedderkopp, N.; Kristensen, P.; Moller, N.C.; Froberg, K.; Cooper, A.R. Cycling to school and cardiovascular risk factors: A longitudinal study. *J. Phys. Act. Health* **2011**, *8*, 1025–1033.
30. Baig, F.; Hameed, M.; Li, M.; Shorthouse, G.; Roalfe, A.; Daley, A. Association between active commuting to school, weight and physical activity status in ethnically diverse adolescents predominately living in deprived communities. *Public Health* **2009**, *123*, 39–41.
31. Borrestad, L.A.B.; Ostergaard, L.; Andersen, L.B.; Bere, E. Associations between active commuting to school and objectively measured physical activity. *J. Phys. Act. Health* **2013**, *10*, 826–832.
32. Brophy, S.; Cooksey, R.; Lyons, R.A.; Thomas, N.E.; Rodgers, S.E.; Gravenor, M.B. Parental factors associated with walking to school and participation in organised activities at age 5: Analysis of the Millennium Cohort Study. *BMC Public Health* **2011**, *11*, doi:10.1186/1471-2458-11-14.
33. Butler, G.P.; Orpana, H.M.; Wiens, A.J. By your own two feet—Factors associated with active transportation in Canada. *Can. J. Public Health* **2007**, *98*, 259–264.
34. Carse, A.; Goodman, A.; Mackett, R.L.; Panter, J.; Ogilvie, D. The factors influencing car use in a cycle-friendly city: The case of Cambridge. *J. Transp. Geogr.* **2013**, *28*, 67–74.
35. Carver, A.; Timperio, A.F.; Hesketh, K.D.; Ridgers, N.D.; Salmon, J.L.; Crawford, D.A. How is active transport associated with children's and adolescents' physical activity over time? *Int. J. Behav. Nutr. Phys. Act.* **2011**, *8*, doi:10.1186/1479-5868-8-126.
36. Cerin, E.; Leslie, E.; Owen, N. Explaining socio-economic status differences in walking for transport: An ecological analysis of individual, social and environmental factors. *Soc. Sci. Med.* **2009**, *68*, 1013–1020.
37. Chillon, P.; Ortega, F.B.; Ruiz, J.R.; De Bourdeaudhuij, I.; Martinez-Gomez, D.; Vicente-Rodriguez, G.; Widhalm, K.; Molnar, D.; Gottrand, F.; Gonzalez-Gross, M.; *et al.* Active commuting and physical activity in adolescents from Europe: Results from the Helena study. *Pediatr. Exerc. Sci.* **2011**, *23*, 207–217.
38. Chillon, P.; Ortega, F.B.; Ruiz, J.R.; Veidebaum, T.; Oja, L.; Maestu, J.; Sjostrom, M. Active commuting to school in children and adolescents: An opportunity to increase physical activity and fitness. *Scand. J. Public Health* **2010**, *38*, 873–879.
39. Cooper, A.R.; Andersen, L.B.; Wedderkopp, N.; Page, A.S.; Froberg, K. Physical activity levels of children who walk, cycle, or are driven to school. *Am. J. Prev. Med.* **2005**, *29*, 179–184.

40. Cooper, A.R.; Page, A.S.; Foster, L.J.; Qahwaji, D. Commuting to school—Are children who walk more physically active? *Amer. J. Prev. Med.* **2003**, *25*, 273–276.
41. Cooper, A.R.; Jago, R.; Southward, E.F.; Page, A.S. Active travel and physical activity across the school transition: The peach project. *Med. Sci. Sports Exerc.* **2012**, *44*, 1890–1897.
42. Cooper, A.R.; Page, A.S.; Wheeler, B.W.; Griew, P.; Davis, L.; Hillsdon, M.; Jago, R. Mapping the walk to school using accelerometry combined with a global positioning system. *Amer. J. Prev. Med.* **2010**, *38*, 178–183.
43. Cooper, A.R.; Wedderkopp, N.; Jago, R.; Kristensen, P.L.; Moller, N.C.; Froberg, K.; Page, A.S.; Andersen, L.B. Longitudinal associations of cycling to school with adolescent fitness. *Prev. Med.* **2008**, *47*, 324–328.
44. Cooper, A.R.; Wedderkopp, N.; Wang, H.; Andersen, L.B.; Froberg, K.; Page, A.S. Active travel to school and cardiovascular fitness in danish children and adolescents. *Med. Sci. Sports Exerc.* **2006**, *38*, 1724–1731.
45. Daly-Smith, A.J.W.; McKenna, J.; Radley, D.; Long, J. The impact of additional weekdays of active commuting to school on children achieving a criterion of 300+minutes of moderate-to-vigorous physical activity. *Health Educ. J.* **2011**, *70*, 428–434.
46. Davis, M.G.; Fox, K.R.; Hillsdon, M.; Coulson, J.C.; Sharp, D.J.; Stathi, A.; Thompson, J.L. Getting out and about in older adults: The nature of daily trips and their association with objectively assessed physical activity. *Int. J. Behav. Nutr. Phys. Act.* **2011**, *8*, doi:10.1186/1479-5868-8-116.
47. de Bruijn, G.J.; Kremers, S.P.J.; Singh, A.; van den Putte, B.; van Mechelen, W. Adult active transportation adding habit strength to the theory of planned behavior. *Am. J. Prev. Med.* **2009**, *36*, 189–194.
48. De Cocker, K.; Cardon, G.; De Bourdeaudhuij, I. Pedometer-determined physical activity and its comparison with the international physical activity questionnaire in a sample of Belgian adults. *Res. Q. Exerc. Sport* **2007**, *78*, 429–437.
49. Dollman, J.; Lewis, N.R. Active transport to school as part of a broader habit of walking and cycling among south Australian youth. *Pediatr. Exerc. Sci.* **2007**, *19*, 436–443.
50. Dombois, O.T.; Braun-Fahrlander, C.; Martin-Diener, E. Comparison of adult physical activity levels in three Swiss alpine communities with varying access to motorized transportation. *Health Place* **2007**, *13*, 757–766.
51. Drygas, W.; Kwasniewska, M.; Kaleta, D.; Pikala, M.; Bielecki, W.; Gluszek, J.; Zdrojewski, T.; Pajak, A.; Kozakiewicz, K.; Broda, G. Epidemiology of physical inactivity in Poland: Prevalence and determinants in a former communist country in socioeconomic transition. *Public Health* **2009**, *123*, 592–597.
52. Ducheyne, F.; De Bourdeaudhuij, I.; Spittaels, H.; Cardon, G. Individual, social and physical environmental correlates of “never” and “always” cycling to school among 10 to 12 year old children living within a 3.0 km distance from school. *Int. J. Behav. Nutr. Phys. Act.* **2012**, *9*, doi:10.1186/1479-5868-9-142.
53. Duncan, E.K.; Scott Duncan, J.; Schofield, G. Pedometer-determined physical activity and active transport in girls. *Int. J. Behav. Nutr. Phys. Act.* **2008**, *5*, doi:10.1186/1479-5868-5-2.

54. Engbers, L.H.; Hendriksen, I.J.M. Characteristics of a population of commuter cyclists in the Netherlands: Perceived barriers and facilitators in the personal, social and physical environment. *Int. J. Behav. Nutr. Phys. Act.* **2010**, *7*, doi:10.1186/1479-5868-7-89.
55. Faulkner, G.; Stone, M.; Buliung, R.; Wong, B.; Mitra, R. School travel and children's physical activity: A cross-sectional study examining the influence of distance. *BMC Public Health* **2013**, *13*, doi:10.1186/1471-2458-13-1166.
56. Ferrar, K.; Olds, T.; Maher, C.; Maddison, R. Time use clusters of New Zealand adolescents are associated with weight status, diet and ethnicity. *Aust. N. Z. J. Public Health* **2013**, *37*, 39–46.
57. Ford, P.; Bailey, R.; Coleman, D.; Woolf-May, K.; Swaine, I. Activity levels, dietary energy intake, and body composition in children who walk to school. *Pediatr. Exerc. Sci.* **2007**, *19*, 393–407.
58. Freeman, L.; Neckerman, K.; Schwartz-Soicher, O.; Quinn, J.; Richards, C.; Bader, M.D.M.; Lovasi, G.; Jack, D.; Weiss, C.; Konty, K.; *et al.* Neighborhood walkability and active travel (walking and cycling) in New York city. *J. Urban Health* **2013**, *90*, 575–585.
59. Fulton, J.E.; Shisler, J.L.; Yore, M.M.; Caspersen, C.J. Active transportation to school: Findings from a national survey. *Res. Q. Exerc. Sport* **2005**, *76*, 352–357.
60. Furie, G.L.; Desai, M.M. Active transportation and cardiovascular disease risk factors in U.S. Adults. *Am. J. Prev. Med.* **2012**, *43*, 621–628.
61. Goodman, A.; Mackett, R.L.; Paskins, J. Activity compensation and activity synergy in British 8–13 year olds. *Prev. Med.* **2011**, *53*, 293–298.
62. Gordon-Larsen, P.; Nelson, M.C.; Beam, K. Associations among active transportation, physical activity, and weight status in young adults. *Obes. Res.* **2005**, *13*, 868–875.
63. Goodman, A.; Brand, C.; Ogilvie, D.; iConnect consortium. Associations of health, physical activity and weight status with motorised travel and transport carbon dioxide emissions: A cross-sectional, observational study. *Environ. Health* **2012**, *11*, doi:10.1186/1476-069X-11-52.
64. Grow, H.M.; Saelens, B.E.; Kerr, J.; Durant, N.H.; Norman, G.J.; Sallis, J.F. Where are youth active? Roles of proximity, active transport, and built environment. *Med. Sci. Sports Exerc.* **2008**, *40*, 2071–2079.
65. Harten, N.; Olds, T. Patterns of active transport in 11–12 year old Australian children. *Aust. N. Z. J. Public Health* **2004**, *28*, 167–172.
66. Hearst, M.O.; *et al.* The relationship of area-level sociodemographic characteristics, household composition and individual-level socioeconomic status on walking behavior among adults. *Transp. Res A Policy Pract.* **2013**, *50*, 149–157.
67. Heelan, K.A.; Donnelly, J.; Jacobsen, D.; Mayo, M.; Washburn, R.; Greene, L. Active commuting to and from school and bmi in elementary school children—Preliminary data. *Child Care Health Dev.* **2005**, *31*, 341–349.
68. Hohepa, M.; Schofield, G.; Kolt, G.S.; Scragg, R.; Garrett, N. Pedometer-determined physical activity levels of adolescents: Differences by age, sex, time of week, and transportation mode to school. *J. Phys. Act. Health* **2008**, *5*, S140–S152.
69. Johnson, T.G.; Brusseau, T.A.; Darst, P.W.; Kulinna, P.H.; White-Taylor, J. Step counts of non-white minority children and youth by gender, grade level, race/ethnicity, and mode of school transportation. *J. Phys. Act. Health* **2010**, *7*, 730–736.

70. King, A.C.; Parkinson, K.N.; Adamson, A.J.; Murray, L.; Besson, H.; Reilly, J.J.; Basterfield, L. Correlates of objectively measured physical activity and sedentary behavior in English children. *Eur. J. Public Health* **2011**, *21*, 424–431.
71. Kwasniewska, M.; Kaczmarczyk-Chalas, K.; Pikala, M.; Broda; Kozakiewicz, K.; Pajak, A.; Tykarski, A.; Zdrojewski, T.; Drygas, W. Socio-demographic and lifestyle correlates of commuting activity in Poland. *Prev. Med.* **2010**, *50*, 257–261.
72. Kwasniewska, M.; Kaczmarczyk-Chalas, K.; Pikala, M.; Broda, G.; Kozakiewicz, K.; Pajak, A.; Tykarski, A.; Zdrojewski, T.; Drygas, W. Commuting physical activity and prevalence of metabolic disorders in Poland. *Prev. Med.* **2010**, *51*, 482–487.
73. Lachapelle, U.; Noland, R.B. Does the commute mode affect the frequency of walking behavior? The public transit link. *Transp. Policy* **2012**, *21*, 26–36.
74. Landsberg, B.; Plachta-Danielzik, S.; Much, D.; Johannsen, M.; Lange, D.; Mueller, M.J. Associations between active commuting to school, fat mass and lifestyle factors in adolescents: The kiel obesity prevention study (kops). *Eur. J. Clin. Nutr.* **2008**, *62*, 739–747.
75. Larouche, R.; Lloyd, M.; Knight, E.; Tremblay, M.S. Relationship between active school transport and body mass index in grades 4-to-6 children. *Pediatr. Exerc. Sci.* **2011**, *23*, 322–330.
76. Leslie, E.; Kremer, P.; Toumbourou, J.W.; Williams, J.W. Gender differences in personal, social and environmental influences on active travel to and from school for Australian adolescents. *J. Sci. Med. Sport* **2010**, *13*, 597–601.
77. Loucaides, C.A.; Plotnikoff, R.C.; Bercovitz, K. Differences in the correlates of physical activity between urban and rural Canadian youth. *J. Sch. Health* **2007**, *77*, 164–170.
78. Marques, E.A.; Pizarro, A.N.; Figueiredo, P.; Mota, J.; Santos, M.P. Modifiable lifestyle behavior patterns, sedentary time and physical activity contexts: A cluster analysis among middle school boys and girls in the salta study. *Prev. Med.* **2013**, *56*, 413–415.
79. Martin, S.L.; Lee, S.M.; Lowry, R. National prevalence and correlates of walking and bicycling to school. *Am. J. Prev. Med.* **2007**, *33*, 98–105.
80. Mendoza, J.A.; Watson, K.; Nguyen, N.; Cerin, E.; Baranowski, T.; Nicklas, T.A. Active commuting to school and association with physical activity and adiposity among US youth. *J. Phys. Act. Health* **2011**, *8*, 488–495.
81. Merom, D.; Miller, Y.D.; van der Ploeg, H.P.; Bauman, A. Predictors of initiating and maintaining active commuting to work using transport and public health perspectives in Australia. *Prev. Med.* **2008**, *47*, 342–346.
82. Metcalf, B.; Voss, L.; Jeffery, A.; Perkins, J.; Wilkin, T. Physical activity cost of the school run: Impact on schoolchildren of being driven to school (earlybird 22). *BMJ Br. Med. J.* **2004**, *329*, 832–833.
83. Molina-Garcia, J.; Castillo, I.; Sallis, J.F. Psychosocial and environmental correlates of active commuting for university students. *Prev. Med.* **2010**, *51*, 136–138.
84. Morckel, V.; Terzano, K. The influence of travel attitudes, commute mode choice, and perceived neighborhood characteristics on physical activity. *J. Phys. Act. Health* **2014**, *11*, 91–98.
85. Mota, J.; Ribeiro, J.C.; Carvalho, J.; Santos, M.P. The physical activity behaviors outside school and bmi in adolescents. *J. Phys. Act. Health* **2010**, *7*, 754–760.

86. Murtagh, E.M.; Murphy, M.H. Active travel to school and physical activity levels of Irish primary schoolchildren. *Pediatr. Exerc. Sci.* **2011**, *23*, 230–236.
87. Naumann, R.B.; Dellinger, A.M.; Anderson, M.L.; Bonomi, A.E.; Rivara, F.P.; Thompson, R.S. Preferred modes of travel among older adults: What factors affect the choice to walk instead of drive? *J. Saf. Res.* **2009**, *40*, 395–398.
88. Nilsson, A.; Andersen, L.B.; Ommundsen, Y.; Froberg, K.; Sardinha, L.B.; Piehl-Aulin, K.; Ekelund, U. Correlates of objectively assessed physical activity and sedentary time in children: A cross-sectional study (the European Youth Heart Study). *BMC Public Health* **2009**, *9*, doi:10.1186/1471-2458-9-322.
89. Oreskovic, N.M.; Sawicki, G.S.; Kinane, T.B.; Winickoff, J.P.; Perrin, J.M. Travel patterns to school among children with asthma. *Clin. Pediatr.* **2009**, *48*, 632–640.
90. Ostergaard, L.; Grontved, A.; Borrestad, L.A.B.; Froberg, K.; Gravesen, M.; Andersen, L.B. Cycling to school is associated with lower bmi and lower odds of being overweight or obese in a large population-based study of Danish adolescents. *J. Phys. Act. Health* **2012**, *9*, 617–625.
91. Ostergaard, L.; Kolle, E.; Steene-Johannessen, J.; Anderssen, S.A.; Andersen, L.B. Cross sectional analysis of the association between mode of school transportation and physical fitness in children and adolescents. *Int. J. Behav. Nutr. Phys. Act.* **2013**, *10*, doi:10.1186/1479-5868-10-91.
92. Owen, C.G.; Nightingale, C.M.; Rudnicka, A.R.; van Sluijs, E.M.F.; Ekelund, U.; Cook, D.G.; Whincup, P.H. Travel to school and physical activity levels in 9–10 year-old UK children of different ethnic origin; Child Heart and Health Study in England (CHASE). *PLoS One* **2012**, *7*, doi: 10.1371/journal.pone.0030932.
93. Pabayo, R.; Maximova, K.; Spence, J.C.; Vander Ploeg, K.; Wu, B.; Veugelers, P.J. The importance of active transportation to and from school for daily physical activity among children. *Prev. Med.* **2012**, *55*, 196–200.
94. Panter, J.; Jones, A.; Van Sluijs, E.; Griffin, S. The influence of distance to school on the associations between active commuting and physical activity. *Pediatr. Exerc. Sci.* **2011**, *23*, 72–86.
95. Rissel, C.; Mulley, C.; Ding, D. Travel mode and physical activity at Sydney university. *Int. J. Environ. Res. Public Health* **2013**, *10*, 3563–3577.
96. Robertson-Wilson, J.E.; Leatherdale, S.T.; Wong, S.L. Social-ecological correlates of active commuting to school among high school students. *J. Adolesc. Health* **2008**, *42*, 486–495.
97. Rosenberg, D.E.; Sallis, J.F.; Conway, T.L.; Cain, K.L.; McKenzie, T.L. Active transportation to school over 2 years in relation to weight status and physical activity. *Obesity* **2006**, *14*, 1771–1776.
98. Roth, M.A.; Millett, C.J.; Mindell, J.S. The contribution of active travel (walking and cycling) in children to overall physical activity levels: A national cross sectional study. *Prev. Med.* **2012**, *54*, 134–139.
99. Sahlqvist, S.; Goodman, A.; Cooper, A.R.; Ogilvie, D. Change in active travel and changes in recreational and total physical activity in adults: Longitudinal findings from the iconnect study. *Int. J. Behav. Nutr. Phys. Act.* **2013**, *10*, doi:10.1186/1479-5868-10-28.

100. Sahlqvist, S.; Song, Y.; Ogilvie, D. Is active travel associated with greater physical activity? The contribution of commuting and non-commuting active travel to total physical activity in adults. *Prev. Med.* **2012**, *55*, 206–211.
101. Sahlqvist, S.; Heesch, K.C. Characteristics of utility cyclists in Queensland, Australia: An examination of the associations between individual, social, and environmental factors and utility cycling. *J. Phys. Act. Health* **2012**, *9*, 818–828.
102. Saksvig, B.I.; Webber, L.S.; Elder, J.P.; Ward, D.; Evenson, K.R.; Dowda, M.; Chae, S.E.; Treuth, M.S. A cross-sectional and longitudinal study of travel by walking before and after school among eighth-grade girls. *J. Adolesc. Health* **2012**, *51*, 608–614.
103. Santos, M.P.; Oliveira, J.; Ribeiro, J.C.; Mota, J. Active travel to school, bmi and participation in organised and non-organised physical activity among Portuguese adolescents. *Prev. Med.* **2009**, *49*, 497–499.
104. Sayers, S.P.; LeMaster, J.W.; Thomas, I.M.; Petroski, G.F.; Ge, B. A walking school bus program impact on physical activity in elementary school children in Columbia, Missouri. *Amer. J. Prev. Med.* **2012**, *43*, S384–S389.
105. Schofield, G.; Badlands, H.; Oliver, M. Objectively-measured physical activity in New Zealand workers. *J. Sci. Med. Sport* **2005**, *8*, 143–151.
106. Sirard, J.R.; Riner, W.F., Jr.; McIver, K.L.; Pate, R.R. Physical activity and active commuting to elementary school. *Med. Sci. Sports Exerc.* **2005**, *37*, 2062–2069.
107. Sisson, S.B.; Tudor-Locke, C. Comparison of cyclists' and motorists' utilitarian physical activity at an urban university. *Prev. Med.* **2008**, *46*, 77–79.
108. Slingerland, M.; Borghouts, L.B.; Hesselink, M.K.C. Physical activity energy expenditure in dutch adolescents: Contribution of active transport to school, physical education, and leisure time activities. *J. Sch. Health* **2012**, *82*, 225–232.
109. Smith, L.; Sahlqvist, S.; Ogilvie, D.; Jones, A.; Corder, K.; Griffin, S.J.; van Sluijs, E. Is a change in mode of travel to school associated with a change in overall physical activity levels in children? Longitudinal results from the SPEEDY Study. *Int. J. Behav. Nutr. Phys. Act.* **2012**, *9*, doi:10.1186/1479-5868-9-134.
110. Smith, L.; Sahlqvist, S.; Ogilvie, D.; Jones, A.; Griffin, S.J.; van Sluijs, E. Is active travel to non-school destinations associated with physical activity in primary school children? *Prev. Med.* **2012**, *54*, 224–228.
111. Spinks, A.; Macpherson, A.; Bain, C.; McClure, R. Determinants of sufficient daily activity in Australian primary school children. *J. Paediatr. Child Health* **2006**, *42*, 674–679.
112. Sugiyama, T.; Merom, D.; Reeves, M.; Leslie, E.; Owen, N. Habitual active transport moderates the association of TV viewing time with body mass index. *J. Phys. Act. Health* **2010**, *7*, 11–16.
113. van Sluijs, E.M.; Fearne, V.A.; Mattocks, C.; Riddoch, C.; Griffin, S.J.; Ness, A. The contribution of active travel to children's physical activity levels: Cross-sectional results from the ALSPAC study. *Prev. Med.* **2009**, *48*, 519–524.
114. Voss, C.; Sandercock, G. Aerobic fitness and mode of travel to school in English schoolchildren. *Med. Sci. Sports Exerc.* **2010**, *42*, 281–287.

115. Wen, L.M.; Merom, D.; Rissel, C.; Simpson, J.M. Weight status, modes of travel to school and screen time: A cross-sectional survey of children aged 10–13 years in Sydney. *Health Promot. J. Austr.* **2010**, *21*, 57–63.
116. Yang, L.; Panter, J.; Griffin, S.J.; Ogilvie, D. Associations between active commuting and physical activity in working adults: Cross-sectional results from the commuting and health in Cambridge study. *Prev. Med.* **2012**, *55*, 453–457.
117. Yelavich, S.; Towns, C.; Burt, R.; Chow, K.; Donohue, R.; Sani, H.S.H.; Taylor, K.; Gray, A.; Eberhart-Phillips, J.; Reeder, A.I. Walking to school: Frequency and predictors among primary school children in Dunedin, New Zealand. *N. Z. Med. J.* **2008**, *121*, 51–58.
118. Chau, J.Y.; van der Ploeg, H.P.; Merom, D.; Chey, T.; Bauman, A.E. Cross-sectional associations between occupational and leisure-time sitting, physical activity and obesity in working adults. *Prev. Med.* **2012**, *54*, 195–200.
119. Dunton, G.F.; Berrigan, D.; Ballard-Barbash, R.; Graubard, B.; Atienza, A.A. Joint associations of physical activity and sedentary behaviors with body mass index: Results from a time use survey of us adults. *Int. J. Obes.* **2009**, *33*, 1427–1436.
120. Granzin, K.L.; Olsen, J.E. Characterizing participants in activities protecting the environment—A focus on donating, recycling, and conservation behaviors. *J. Public Policy Mark.* **1991**, *10*, 1–27.
121. Mota, J.; Gomes, H.; Almeida, M.; Ribeiro, J.C.; Carvalho, J.; Santos, M.P. Active *versus* passive transportation to school—differences in screen time, socio-economic position and perceived environmental characteristics in adolescent girls. *Ann. Hum. Biol.* **2007**, *34*, 273–282.
122. Wong, B.Y.M.; Faulkner, G.; Buliung, R.; Irving, H. Mode shifting in school travel mode: Examining the prevalence and correlates of active school transport in Ontario, Canada. *BMC Public Health* **2011**, *11*, doi:10.1186/1471-2458-11-618.
123. Wang, Y.; Tuomilehto, J.; Jousilahti, P.; Antikainen, R.; Mahonen, M.; Katzmarzyk, P.T.; Hu, G. Occupational, commuting, and leisure-time physical activity in relation to heart failure among finnish men and women. *J. Am. Coll. Cardiol.* **2010**, *56*, 1140–1148.
124. Besser, L.M.; Dannenberg, A.L. Walking to public transit steps to help meet physical activity recommendations. *Am. J. Prev. Med.* **2005**, *29*, 273–280.
125. Cerin, E.; Leslie, E.; du Toit, L.; Owen, N.; Frank, L.D. Destinations that matter: Associations with walking for transport. *Health Place* **2007**, *13*, 713–724.
126. Dolnicar, S.; Laesser, C.; Matus, K. Short-haul city travel is truly environmentally sustainable. *Tour. Manag.* **2010**, *31*, 505–512.
127. Edwards, R.D. Public transit, obesity, and medical costs: Assessing the magnitudes. *Prev. Med.* **2008**, *46*, 14–21.
128. Goetzke, F.; Rave, T. Bicycle use in Germany: Explaining differences between municipalities with social network effects. *Urban Stud.* **2011**, *48*, 427–437.
129. Lachapelle, U.; Frank, L.; Saelens, B.E.; Sallis, J.F.; Conway, T.L. Commuting by public transit and physical activity: Where you live, where you work, and how you get there. *J. Phys. Act. Health* **2011**, *8*, S72–S82.

130. Lachapelle, U.; Frank, L.D. Transit and health: Mode of transport, employer-sponsored public transit pass programs, and physical activity. *J. Public Health Policy* **2009**, *30*, S73–S94.
131. Lee, C. Environment and active living: The roles of health risk and economic factors. *Am. J. Health Promot.* **2007**, *21*, 293–304.
132. MacDonald, J.M.; Stokes, R.J.; Cohen, D.A.; Kofner, A.; Ridgeway, G.K. The effect of light rail transit on body mass index and physical activity. *Amer. J. Prev. Med.* **2010**, *39*, 105–112.
133. Moudon, A.V.; Chanam Lee, C.; Cheadle, A.D.; Collier, C.W.; Johnson, D.; Schmid, T.L.; Weather. R.D. Cycling and the built environment, a US perspective. *Transp Res. D Transp. Environ.* **2005**, *10*, 245–261.
134. Villanueva, K.; Giles-Corti, B.; McCormack, G. Achieving 10,000 steps: A comparison of public transport users and drivers in a university setting. *Prev. Med.* **2008**, *47*, 338–341.
135. Wasfi, R.A.; Ross, N.A.; El-Geneidy, A.M. Achieving recommended daily physical activity levels through commuting by public transportation: Unpacking individual and contextual influences. *Health Place* **2013**, *23*, 18–25.
136. Wener, R.E.; Evans, G.W. A morning stroll—levels of physical activity in car and mass transit commuting. *Environ. Behav.* **2007**, *39*, 62–74.
137. Fuller, D.; Cummins, S.; Matthews, S.A. Does transportation mode modify associations between distance to food store, fruit and vegetable consumption, and bmi in low-income neighborhoods? *Am. J. Clin. Nutr.* **2013**, *97*, 167–172.
138. Lemstra, M.; Rogers, M.; Thompson, A.; Moraros, J. Prevalence and correlates of physical activity within on-reserve first nations youth. *J. Phys. Act. Health* **2013**, *10*, 430–436.
139. Swanson, K.C.; McCormack, G.R. The relations between driving behavior, physical activity, and weight status among Canadian adults. *J. Phys. Act. Health* **2012**, *9*, 352–359.
140. Wen, L.; Orr, N.; Millett, C.; Rissel, C. Driving to work and overweight and obesity: Findings from the 2003 New South Wales Health Survey, Australia. *Int. J. Obes.* **2006**, *30*, 782–786.
141. Wennberg, P.; Wensley, F.; Johansson, L.; Boman, K.; Di Angelantonio, E.; Rumley, A.; Lowe, G.; Hallmans, G.; Jansson, J.-H. Reduced risk of myocardial infarction related to active commuting: Inflammatory and haemostatic effects are potential major mediating mechanisms. *Eur. J. Cardiovasc. Prev. Rehabil.* **2010**, *17*, 56–62.
142. Kesse-Guyot, E.; Peneau, S.; Mejean, C.; Szabo de Edelenyi, F.; Galan, P.; Hercberg, S.; Lairon, D. Profiles of organic food consumers in a large sample of french adults: Results from the Nutrinet-Sante cohort study. *PLoS One* **2013**, *8*, doi:10.1371/journal.pone.0076998.
143. Magnusson, M.K.; Arvola, A.; Hursti, U.-K.K.; Aberg, L.; Sjoden, P.-O. Choice of organic foods is related to perceived consequences for human health and to environmentally friendly behavior. *Appetite* **2003**, *40*, 109–117.
144. Miao, L.; Wei, W. Consumers' pro-environmental behavior and the underlying motivations: A comparison between household and hotel settings. *Int. J. Hospitality Manag.* **2013**, *32*, 102–112.
145. Nie, C.; Zepeda, L. Lifestyle segmentation of US food shoppers to examine organic and local food consumption. *Appetite* **2011**, *57*, 28–37.

146. Petersen, S.B.; Rasmussen, M.A.; Strom, M.; Halldorsson, T.I.; Olsen, S.F. Sociodemographic characteristics and food habits of organic consumers—A study from the Danish national birth cohort. *Public Health Nutr.* **2013**, *16*, 1810–1819.
147. Thogersen, J.; Olander, F. To what degree are environmentally beneficial choices reflective of a general conservation stance? *Environ. Behav.* **2006**, *38*, 550–569.
148. Torjusen, H.; Brantsaeter, A.L.; Haugen, M.; Lieblein, G.; Stigum, H.; Roos, G.; Holmboe-Ottesen, G.; Meltzer, H.M. Characteristics associated with organic food consumption during pregnancy; data from a large cohort of pregnant women in Norway. *BMC Public Health* **2010**, *10*, doi:10.1186/1471-2458-10-775.
149. Torjusen, H.; Lieblein, G.; Naes, T.; Haugen, M.; Meltzer, H.M.; Brantsaeter, A.L. Food patterns and dietary quality associated with organic food consumption during pregnancy; data from a large cohort of pregnant women in Norway. *BMC Public Health* **2012**, *12*, doi:10.1186/1471-2458-12-612.
150. Burgess, C.P.; Johnston, F.H.; Berry, H.L.; McDonnell, J.; Yibarbuk, D.; Gunabarra, C.; Mileran, A.; Bailie, R.S. Healthy country, healthy people: The relationship between indigenous health status and "caring for country". *Med. J. Australia* **2009**, *190*, 567–572.
151. Litt, J.S.; Soobader, M.J.; Turbin, M.S.; Hale, J.W.; Buchenau, M.; Marshall, J.A. The influence of social involvement, neighborhood aesthetics, and community garden participation on fruit and vegetable consumption. *Amer. J. Public Health* **2011**, *101*, 1466–1473.
152. MacMillan Uribe, A.L.; Winham, D.M.; Wharton, C.M. Community supported agriculture membership in Arizona. An exploratory study of food and sustainability behaviors. *Appetite* **2012**, *59*, 431–436.
153. Maples, M.; Morgan, K.L.; Interis, M.G.; Harri, A. Who buys food directly from producers in the southeastern United States? *J. Agric. Appl. Econ.* **2013**, *45*, 509–518.
154. Kamphuis, C.B.M.; Giskes, K.; de Bruijn, G.-J.; Wendel-Vos, W.; Brug, J.; van Lenthe, F.J. Environmental determinants of fruit and vegetable consumption among adults: A systematic review. *Br. J. Nutr.* **2006**, *96*, 620–635.
155. Baik, J.-Y.; Lee, H. Habitual plate-waste of 6- to 9-year-olds may not be associated with lower nutritional needs or taste acuity, but undesirable dietary factors. *Nutr. Res.* **2009**, *29*, 831–838.
156. Marlette, M.A.; Templeton, S.B.; Panemangalore, M. Food type, food preparation, and competitive food purchases impact school lunch plate waste by sixth-grade students. *J. Amer. Diet. Assoc.* **2005**, *105*, 1779–1782.

© 2015 by the authors; licensee MDPI, Basel, Switzerland. This article is an open access article distributed under the terms and conditions of the Creative Commons Attribution license (http://creativecommons.org/licenses/by/4.0/).
